# Comprehensive approach for predicting toxicological effects of ionic liquids on several biological systems using unified descriptors

**DOI:** 10.1038/srep33403

**Published:** 2016-09-14

**Authors:** Chul-Woong Cho, Stefan Stolte, Yeoung-Sang Yun

**Affiliations:** 1 School of Chemical Engineering, Chonbuk National University, 567 Beakje-dearo, Deokjin-gu, Jeonju, Jeonbuk 561-756, South Korea; 2 Centre for Environmental Research and Sustainable Technology (UFT), University of Bremen, Leobener Straße, 28359, Bremen, Germany; 3 Department of Environmental Analysis, Faculty of Chemistry, University of Gdaňsk sk ul, Wita Stwosza 63, 80-308, Gdaňsk, Poland

## Abstract

The challenge and opportunity for design of environmentally-benign ionic liquids (ILs) would start from prediction of their toxicological effects on several endpoints solely based on the structural formulas. Especially, a comprehensive yet simple equation able to predict several biological responses to IL toxicity is of much advantage. Therefore, based on 50 toxicity testing systems on ILs a comprehensively approachable prediction method was developed. For the modelling, approximately 1600 toxicity values measured by several biological systems and an amended linear free energy relationship (LFER) model were used. Since the toxicological activities of an IL could be differently described according to sensitivity of toxicity testing systems, the sensitivity of each of toxicity testing systems was also estimated in the modelling. By statistical analysis with the calculated descriptors, a LFER model was built. Also the sensitivity value of each system on the basis of the comprehensively approachable model was numerically estimated. In results, it was observed that the combination of single model and sensitivity terms was able to predict each of 50 toxicological effects of ILs with R^2^ of 0.593~0.978, and SE of 0.098~0.699 log unit, and the total data set with R^2^ of 0.901 and SE of 0.426 log unit.

Ionic liquids (ILs) as a molten salt around room temperature have been highlighted in chemical industries because of their excellent physicochemical properties^1^. Especially, their replaceability of traditional organic solvents causing environmental pollutions makes them more interesting in green or sustainable chemistry^2^. Moreover, since they have uncountable structural diversities, the potentials of their application may be boundless. In detailed information on the properties and application of ILs is introduced by review article^3^.

On the other hand, ILs can play a role as toxicant in the viewpoint of toxicology, and will continue to impact on the environment due to their structural stability. Therefore, toxicological natures of ILs should be well clarified before released into the environments. So far, millions of ILs structures have been proposed and several thousands are described in literature. However, the toxicological testing of such large substance sets would be labor intensive, time and material consuming; it may be limited to all types of ILs. For extensive testing and proactive designing of the numerous IL structures, computational modelling is desirable as an alternative of experimental determinations because it is faster, safer, and less expensive. Indeed, OECD^4^ and REACH^5^ recommend the approach like quantitative structure activity relationship (QSAR) for risk management. With the motivation, several researchers have developed the theoretical models for the toxicological effects of ILs on various toxicity testing systems including water fleas^6^,^7^,^8^,^9^,^10^,^11^,^12^,^13^,^14^.algae^12^,^15^,^16^, animal cell^12^,^15^,^17^,^18^,^19^, bacteria^12^,^15^,^20^,^21^,^22^,^23^,^24^,^25^,^26^,^27^, and enzyme activity^12^,^17^,^28^,^29^,^30^,^31^,^32^. However, since the previous models have physico-chemically ambiguous parameters and each study employs different parameters according to the toxicity testing systems, it is hard to comprehensively understand the structural effects of ILs on their toxicological activities to various environmental organisms. Moreover, QSAR models for some responses e.g., algal photosynthetic activity and Hela cell lines’ growth have not been developed due to the lack of experimental toxicity data. Therefore, to complement these shortcomings, a simple linear model based on 50 types of biological responses with about 1600 data points on the toxicity of ILs was developed using unified parameters which are linear free energy relationship (LFER) descriptors. In the viewpoint of finding relationships among biological responses, the concept of this research is slightly related to previous article^33^, which presented that based on quantitative toxicity-toxicity relationship (QTTR), interspecies correlation between different biological responses to toxicants can be applied to predict non-existing toxicity data for a particular compound. However, the QTTR model requires experimentally measured toxicity values, and moreover it could not address the toxicological mechanisms in molecular basis. Therefore, in this study, the employed LFER descriptors as unified parameters were calculated based on *in silico* methods i.e., density functional theory (DFT)^34^, conductor-like screening (COSMO) model^35^, and obprop internet freeware^36^.

## Explanation on theoretical model

For modelling, linear free energy relationship (LFER) concept was used because it consists of simple and well-defined solute descriptors (E, S, A, B, V, J^−^, J^+^)^37^ and it has been used for predicting the toxicological effects of chemicals as previously shown^38^. The LFER model is:


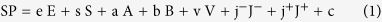


where, SP stands for solute property. The small letters (e, s, a, b, v, j^−^, j^+^, and c) are system parameters (sometimes, called as system coefficients) explaining the molecular interactions of toxicity testing system. They can be simply determined by multiple linear regression (MLR) analysis. And the capital letters are solute descriptors that describe the intrinsic molecular interaction potentials of an atom or a molecule. The meanings of the solute descriptors of a molecule or an atom are as following: E [cm^3^ mol^−1^/10] – excess molar refraction due to interaction of n- or pi- electron lone pairs; S [dimensionless] – dipolarity/polarizability by dipole-dipole and dipole-induced dipole interactions; A and B [dimensionless] – hydrogen bonding acidity and hydrogen bonding basicity; V [cm^3^ mol^−1^/100] – McGowan volume; J^−^ and J^+^ [dimensionless] – ionic interactions of the anion and the cation, respectively.

Since the prediction modelling was performed in the assumption of ion dissociation status, comprised of two ions i.e., cation and anion, each terms were divided into cationic and anionic part as Eq. (2).


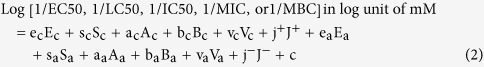


where, the solute property includes five endpoints i.e., half maximal effective concentration (EC50), half maximal lethal concentration (LC50), half maximal inhibitory concentration (IC50), minimal inhibitory concentration (MIC) and minimal biocidal concentration (MBC) in the log units of mM. For the positive trends of system parameters on IL toxicity, the five end points were transferred into inverted logarithm e.g., log 1/EC50. The subscripts ‘c’ and ‘a’ means cation and anion, respectively. Here, the experimental values for LFER descriptors are not limited to all ions of ILs; thus they were calculated according to the methods developed by our group^39^.

## Results and Discussion

To build the comprehensively approachable model based on various biological responses to IL toxicity, it was hypothesized that toxicity test methods have a similar response pattern and different sensitivities according to IL chemical structure and organism’s tolerance to toxicants. However, if the hypothesis is inconsistent to some toxic compound with specific action and to some testing method occupied by different molecular interactions, it shows large prediction error and/or low coefficient of determination in statistical analysis.

In order to address the degree of the sensitivity of each method based on Eq. (2), we simply amended the LFER model by adding a term z_x_Z_x_ as shown Eq. (3).


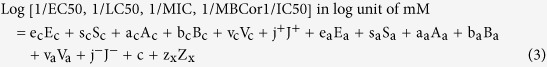


where, subscript ‘x’ of Z term indicates kinds (1~58) of the toxicity testing methods (see Table 1). Initially, the system parameters of the model and sensitivity terms based on toxicity values of 52 methods (No.1~52 in Table 1) were studied and the rest i.e., six systems (No. 53~58 in Table 1) was used for an example study. When performing MLR analysis, each Z_x_ should have fixed value as +1 for a specific case while the rest should be zero (see an example in Table S2). The system parameter z means degree of toxicity testing system’s sensitivity. It can be mathematically calculated as an average of the differences between predicted values and experimental values in a specific system; but it can be automatically determined by MLR. The other system parameters of Eq. (3) (i.e., e_c_, s_c_, a_c_, b_c_, v_c_, j^+^, e_a_, s_a_, a_a_, b_a_, v_a_, j^−^ and c) can be determined by the same step i.e., MLR. In the statistical analysis, the importance of each term for Eq. (3) was also checked for simplifying the model. The degree of the significance was judged by their probability values (p-values) estimated by MLR. Here, if the p-value is higher than 0.05, it is not significant; thus it was excluded, while if lower, it is significant and included for a model. From the analysis, it was found that three descriptors in the anion part i.e., S_a_, A_a_, and B_a_ were useless as shown that they have higher p-values than 0.05, while all cation-related terms and rests of anionic terms (E_a_, V_a_ and J^−^) have reasonable contributions to the model because they have lower p-value than 0.05. The determined system parameters of eight selected terms that can explain a common toxic effect of ILs are as below:


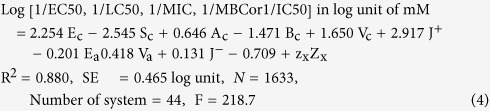


where, the system parameters including z_x_ values were estimated based on 1633 data points of 44 toxicity testing systems. The R^2^ value is 0.880 and standard error is 0.465 log unit, which indicate reasonable accuracy of Eq. (4). The z values of each testing methods are given in Table 1. In Table 1, z value of *leukemia rat cell line* was zero because it automatically became a standard test method due to that the method has the largest number of data points among the studied systems. Here, the magnitude of z value indicates the degree of sensitivity, that is, the higher value means the higher sensitivity. The sensitivity values are given in Table 1.

In the selection of biological responses to build Eq. (4), the acceptable value of R^2^ was internally set to 0.6. However, in case of MBC of *R. rubra* (32), its R^2^ is slightly lower than 0.6 and its standard error is low i.e., 0.34 log unit. Thus we included MBC of *R. rubra* for building Eq. (4). Among the initially studied 52 methods, eight cases (No. 45~52 in Table 1) were exceptional cases as shown their large prediction errors. Those are acetylcholinesterase (45), *L. minor* (46), *P. subcapitata* (47), MBC of *P. vulgaris* (48), MIC of *P. aeruginosa* (49), MBC of *P. aeruginosa* (50), MIC of *S. marcescens* (51), and MBC of *S. marcescens* (52). When applying the Eq. (4) to predict log 1/EC50 values of acetylcholinesterase, large distributions of data points in a correlation between calculated and observed values were observed (see No. 45 in Figure S2). It might be due to that the toxicological interactions of ILs to the enzyme inhibition were different from other studies (No 1~44 in Table 1). Since there are not enough data points or experimental data distributions are narrow, no. 46~52 (*L. minor* (46) and antimicrobial testing methods i.e., MBC of *P. vulgaris* (48), MIC of *P. aeruginosa* (49), MBC of *P. aeruginosa* (50), MIC of *S. marcesene* (51), and MBC of *S. marcesene* (52) could not be predicted by Eq. (4) (see No. 46, 48~52 in Figure S2). In case of growth rate of *P. subcupitata* (48), the correlation by Eq. (4) was dependent on dataset from two different research groups i.e., Prof. Yun and coworkers^40^,^41^,^42^ and Prof. Pretti and coworkers^43^. Actually, the calculated values by Eq. (4) for the former case were well correlated with observed ones with R^2^ of 0.857, while those of the latter case were scattered in the correlation (see Figure S3). The exceptional cases from application domain of Eq. (4) should be individually developed. Additionally, some outliers were observed and given in supporting information 2. Generally, the outliers are large cationic molecules with long alkyl chains e.g., trihexyldecylphosphonium [P666-10]^+^, trihexyldodecylphosphonium [P666-12]^+^, and trihexylbutadecylphosphonium [P666-14]^+^. It was guessed that their steric effects and low water-solubility lead to lower toxic effect than those calculated by Eq. (4).

As expected, the correlation between observed and calculated toxicity values by Eq. (4) of a specific system has rather different a linear slope (see Figure S1), because each toxicity testing system have different tolerances according to IL chemical structures. Nevertheless, their linear directions or trends of data distribution between observed and calculated values were similar as we hypothesized at the beginning of this study. Their z values were mathematically calculated as an average value of the differences between measured and calculated log 1/EC50 by Eq. (4). And each linear slope (α_x_) and constant (*β*_x_) between observed and calculated values by Eq. (4) were determined by linearly fitting using Sigma plot and the values are given in Table 1. Additionally their fittings are shown in Figure S1 of Supplementary information 1.

### The comprehensively approachable prediction method provides several advantages

First, it can simplify the toxicological meanings of several minimal toxicity of ILs using nine descriptors and three sensitive terms as Eq. (4). However, as some outliners and exceptional testing systems (45~52 in Table 1) from Eq. (4) were demonstrated, the prediction method cannot perfectly replace the experimental estimations. Nevertheless, it is helpful when doing the experimental design. Again, it is expected that Eq. (4) can be used as a starting point for estimating of toxic effects of ILs towards different endpoints of environmental organisms.

Second, the explanation on toxicological interactions of IL cation and anion identified by the model can facilitate design of new IL structures or selection of IL ions by a combination of cation and anion which already exist. Especially, the toxic effect of anion, which is one of issues in the estimation of IL toxicity, was clearly explained by three terms i.e., E_a_, V_a_, and J^−^. The three system parameters describe that as increasing McGowan volume and ionic interaction of anion, IL toxicity increases, while an increase of excess molar refraction of anion leads to decrease in IL toxicity. Based on anionic part in Eq. (4) i.e., −0.201 E_a_ + 0.418 V_a_ + 0.131 J^−^, the magnitude order of 57 anions’ toxic effect can be arranged (Table S3). Note that additional effects causing increase or decrease of anion toxicity e.g., hydrolysis or biodegradability were not considered. Similarly, the toxic magnitude of cations can be calculated by cationic part of Eq. (4) (2.254 E_c_ − 2.545 S_c_ + 0.646 A_c_ − 1.471 B_c_ + 1.650 V_c_ + 2.917 J^+^) as given in Table S4. In the viewpoint of molecular interactions in IL toxicity, the contribution of McGowan volume and charge interactions of cation has the same trend with those of V_a_ and J^−^, while E_c_ is opposite to E_a_ because in general E_a_ have near zero or negative values unlikely those of cations. As seen in Eq. (4), cation needs more descriptors (i.e., S_c_, A_c_ and B_c_) than anion. The H-bonding acidity term of cation (A_c_) is proportional to the toxic effect of ILs, while H-bonding dipolarity/polarizability and H-bonding basicity terms of cations lead to a reduction of the toxicity.

Third, degree of the biological inhibition of ILs in toxicity test methods can be numerically explained by sensitivity-related terms i.e., z_x_, *α*_x_ and *β*_x_ shown in Table 1. It will be helpful to understand environmental aspects of ILs and select ILs for appropriate use e.g., excellent antimicrobial activity in medicinal applications or low toxic action in sustainable chemistry.

**Figure 1:**
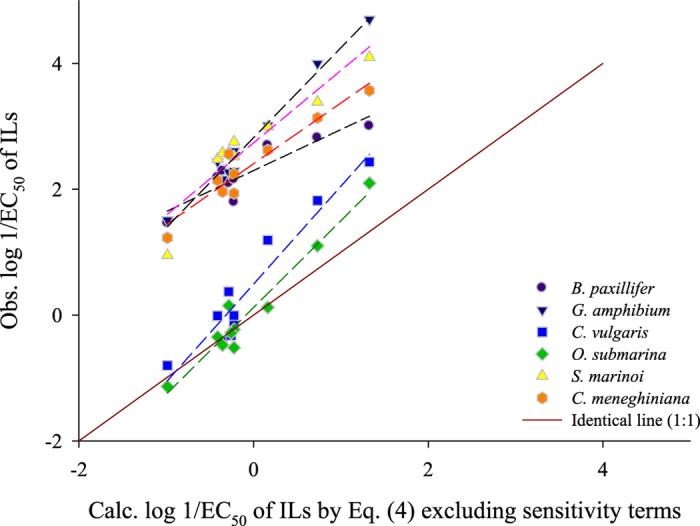
Correlations between observed log 1/EC_50_ of ILs to *growth rate* of algae species and calculated ones by the comprehensive model.

Fourth, the calculated value by Eq. (4) can be used as an indicator to correlate the predicted values with toxicity values from new toxicity testing system. Surely, it should need small number of data set to determine sensitivity related terms i.e., *z*_x_, *α*_x_ and *β*_x_. Unlike real modelling steps where plenty of data points are required, the calculated value by Eq. (4) (2.254 E_c_ − 2.545 S_c_ + 0.646 A_c_ − 1.471 B_c_ + 1.650 V_c_ + 2.917 J^+^ − 0.201 E_a_ + 0.418 V_a_ + 0.131 J^−^ − 0.709) can simply correlate with a few experimental dataset. For a validation test, some examples were made by correlating toxicity values from six algae species i.e., *B. paxillifer*, *G. amphibium*, *C. vulgaris*, *O. submarina*, *S. marinoi*, and *C. meneghiniana*. Each species has 10 toxicity values measured by Latała *et al*^44^,^45^. In results, the calculated values of ILs by Eq. (4) are well correlated with observed ones with R^2^ of 0.82~0.98 (Fig. 1). Their sensitivity terms were estimated and are given in 53~58 of Table 1. For validating the predicted values of test set, the mean absolute error (MAE) criteria derived by Roy *et al.*^46^. was used since R^2^-based metrics is sometimes strongly dependent on the distribution of data. The MAE based estimation can be performed based on an internet freeware tool ‘XternalValidationPlus’ (available at http://dtcab.webs.com/software). Detailed information including theoretical background is given in the article by Roy *et al.*^46^. The validation result using the MAE based criteria in the condition after removing 5% data with high deviations showed MAE (95% data) of 0.2302 with an indication of a good predictability.

Since each fitting between observed and calculated values of ILs has different slope (*α*_x_) and constant (*β*_x_), the model can be expressed as below:


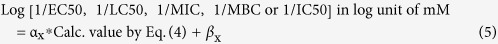


**Figure 2:**
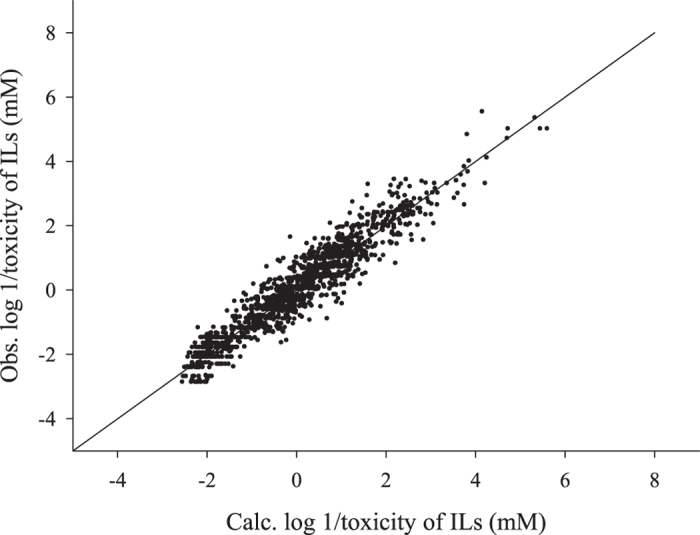
A correlation between the calculated [by Eq. (5)] and the observed toxicity values of ILs to 50 testing methods.

In result, the calculated values by Eq. (5) have an enhanced agreement with the measured toxicity values with R^2^ of 0.901 and SE of 0.426 log unit, compared to those by Eq. (4). The fitting by Eq. (5) is shown in Fig. 2.

## Conclusions

In this study, for the first time we correlated numerous toxicity data (with around 8 orders of magnitudes) of ILs (comprising of around 250 cations and 60 anions) in 58 systems to unified physicochemical (LFER) descriptors and developed a comprehensively approachable prediction method. It is therefore possible to make predictions on the toxicity to a certain test system, or even species sensitivity distributions, for nearly all possible ionic salt combinations. Moreover, by performing the modelling, the contribution degree of molecular interaction potentials of IL cation and anion to various toxicological responses was numerically estimated, and the sensitivity (including tolerance) of each testing system was valued. The prediction model includes excess molar refraction, McGowan volume, and charge interaction of cation and anion, dipolarity/polarizability, H-boding acidity and basicity of cation. It is indicating that ILs may act as separated ions. The magnitude of six terms (E_c_, A_c_, V_c_, J^+^, V_a_, and J^−^) related to an increases of IL toxicity, while that of the rests terms (S_c_, B_c_, and E_a_) lead to a decrease.

It is expected that the comprehensively approachable method will provide faster, and safer toxicity estimation compared to experimental performances; thus it will be useful to efficiently manage numerous types of ILs and to design eco-friendly IL structure. Nevertheless, it needs to be further studied for check of a range of its application domains and simplifying the prediction model. Actually the Eq. (4) for intrinsic values of IL ions in a specific toxicity testing method has nine terms excluding sensitivity terms (z, *α*_*x*_, and *β*_*x*_). Moreover, since the validation of the prediction steps via Eq. (4) and Eq. (5) was performed with only growth rates of six algae species, external validation and reliability should be further examined. Furthermore, modelling for exceptional cases (e.g., acetylcholinesterase inhibition) should be made using the same descriptors to help us understand their toxic mechanisms based on the same chemical meanings.

## Experimental

### Database of ionic liquids and their abbreviations

For model development and some example study for the validation, half maximal effective concentration (EC50), half maximal lethal concentration (LC50), half maximal inhibitory concentration (IC50), minimum inhibitory concentration (MIC) and minimum biocidal concentration (MBC) values of ILs around 2200 data points towards 58 toxicity testing batteries (as given Table 1) were collected from literatures^6^,^20^,^21^,^22^,^39^,^40^,^41^,^42^,^43^,^44^,^45^,^47^,^48^,^49^,^50^,^51^,^52^,^53^,^54^,^55^,^56^,^57^,^58^,^59^,^60^,^61^,^62^,^63^,^64^,^65^,^66^,^67^,^68^,^69^,^70^,^71^. The selected ILs are comprised of several head groups (i.e., piperidinium, sulfonium, melamine, morphodinium, guadinium, ammonium, phosphonium, imidazolium, pyridinium, quinolinium, and purinium with differently functionalized substitutes); which are around 200 types of cations, and 57 types of anions. The lists and abbreviations of IL ions were given in Supplementary information 1. And the collected data set were given in supporting information 2.

### The studied toxicity testing methods for modeling

As listed in Table 1, total 58 biological responses to ILs’ toxicity for modelling were studied as below:

[Cell line] - Viability of three different animal cells such as leukemia rat IPC-81, MCF-7, Hela cell in different experiment conditions i.e., incubation time (24 h or 48 h) or the presence and absence of 10 percent foetal bovine serum (FBS);

[Algae] - Growth rates tests of eight algal species i.e., *Scenedesmus vacuolatus, Pseudokirchneriella subcapitata, Bacillaria paxillifer, Geitlerinema amphibium, Chlorella vulgaris, Oocystis submarina, Skeletonema marino, Cycolotella meneghiniana*; and photosynthetic activity of *P. subcapitata*;

[Water flea] - Immobilization to *Daphnia magna*; [Enzyme] - Inhibition of acetylcholinesterase activity; [Duckweed] - Growth response of *Lamina minor*;

[Bacteria and fungi] - Inhibition of luminescence (*Vibrio fischeri*); growth rate of gram-positive (*Listeria monocytogenes* L4 and *Staphylococcus aureus* S244) and gram-negative bacteria (*Escherichia coli* E149 and *Aeromonas hydrophilia* A97); and antimicrobial properties i.e., MIC and MBC of ILs [Rod type] *Pseudomonas aeruginosa* NCTN 6749, *E.coli* ATCC 25922, *Proteus vulgaris* NCTC 4635, *Klebsiella pneumoniae* ATCC 33495, *Salmonella enteriditis, Listeria monocytogenes, Serratia marcescens* ATCC 8100; [Bacillus type] *Bacillus subtillis* ATCC 6633; [Cocci type] *Staphylococcus epidermidis* ATCC 12228, *Staphylococcus aureus* ATCC 6538, *S. taphulococcus aureus* (MRSA) ATCC 43300, *Enterococcus hirae* ATCC 10541, *Micrococcus luteus* ATCC 9341, *Enterococcus faecalis* ATCC 29212, *Moraxella catarrhalis* ATCC 25238; [Yeast like fungi] - *Candida albicans* ATCC 10231, *Rhodotorula rubra* PhB, *Candida glabrata* DMS 11226, *Candida tropicalis* KKP 334, *Saccharomyces cerevisiae* ATCC9763, *Saccharomyces cerevisiae* JG, *Saccharomyces cerevisiae* JGCDR1, *Geotrichum candidum*, and *Rhodotorula mucilaginosa*.

### Modelling method

Modelling steps can be shortly described as below:

1. Theoretical model was developed based on LFER concept (Eq. 2) where the descriptors of cation and anion were separately provided since two ions may act in dissociated status. In the model, a parameter was further added as a dummy variable Z (i.e., +1) of each testing method for estimating sensitivity coefficient (z) as Eq. (3). (see Table S2).
2. After inserting the calculated descriptors of cations & anions and dummy variable of each biological system to Eq. (3), multiple linear regression was performed to analyze the relevance of the employed descriptors to toxicity values, by checking their *p*-value. After excluding useless descriptors with higher *p*-value than 0.05, MLR analysis was again performed. From this step, the system coefficients of the selected descriptors and the sensitivity value (*z*) of each method were determined as Eq. (4).
3. Based on the system coefficients including z of Eq. (4), an intrinsic value of ILs in specific toxicity testing system was calculated.
4. The calculated intrinsic values of ILs were correlated with experimentally measured toxicity values to determine the sensitivity-related terms (*α*_x_ and *β*_x_).

### Computational details

For calculation of LFER descriptors, several sub-parameters are needed. To obtain the subparameters, targeted IL structures are calculated by using density functional theory (DFT)^34^ and conductor-like screening model (COSMO)^35^ in the Turbomole program package (version 5.10)^72^. First, reasonably starting IL structure were optimized by calculation of (RI-)BP86/SV(P)^73^,^74^,^75^,^76^ in gas phase. The vibration frequencies of each structure were calculated by using AOFORCE^77^,^78^. They were further refined with the TZVP basis set^79^, and then a full optimization was performed with inclusion of COSMO^35^. Those calculations gave us the.ccf of each IL structure, and the files were read to obtain the sub-parameters by COSMO-RS^80^ based on BP-TZVP-C21-0108 parameterization. For molecular reflectivity of molecule, we used obprop internet freeware^36^. The chemical meaning of sub-parameters and the calculation methods for LFER descriptors using the calculated sub-parameters are given in Supplementary information 1. The values are given in supporting information 2 (excel file).

### Statistical analysis

Multiple linear regressions were performed by SPSS 12.0.1 for Windows, and fitting was done by Sigma-Plot for Windows versions 10.0.

## Additional Information

**How to cite this article**: Cho, C.-W. *et al.* Comprehensive approach for predicting toxicological effects of ionic liquids on several biological systems using unified descriptors. *Sci. Rep.* **6**, 33403; doi: 10.1038/srep33403 (2016).

## Supplementary Material

Supplementary Information 1

Supplementary Information 2

## Figures and Tables

**Table 1:** The studied biological systems (ends point) and data number (N) for modelling.

NO.	Biological system (end point)	R^2^	SE	*z*_*x*_	*α*_*x*_	*β*_*x*_	N
1	*Leukemia rat cell* (A)	0.73	0.47	0.00	1.00	0.00	199
2	*S. vacuolatus* (A)	0.75	0.70	1.45	1.12	−0.18	48
3	*V. fischeri* (A)	0.69	0.35	0.23	1.38	−0.47	108
4	*D. magna* (B & C)	0.81	0.42	1.63	1.02	−0.04	70
5	PA^II^ of *P.subcapitata* (A)	0.68	0.33	−0.73	1.43	0.29	8
6	MCF-7 (C)	0.81	0.29	−0.05	1.80	−0.38	16
7	*Hela* cell with FBS for 24 h (A)	0.67	0.43	−0.34	0.95	−0.03	23
8	*Hela* cell without FBS for 24 h (A)	0.85	0.30	−0.02	1.40	0.12	17
9	*Hela* cell for 48 h (A)	0.76	0.33	−0.26	0.78	−0.08	38
10	GR^III^ of *A. hydrophila* (A)	0.86	0.32	−1.00	1.07	0.04	25
11	GR^III^ of *E. coli* (A)	0.85	0.32	−0.98	1.08	0.04	25
12	GR^III^ of *L. monocytogenes* (A)	0.86	0.32	−1.02	1.06	0.03	25
13	GR^III^ of *S. aureus* (A)	0.85	0.33	−0.97	1.07	0.35	25
14	*E. coli* (E)	0.87	0.49	−0.88	0.90	−0.15	91
15	*E. coli* (F)	0.93	0.42	−1.13	0.90	−0.06	45
16	*M. luteus* (E)	0.80	0.43	−0.55	1.00	0.00	46
17	*M. luteus* (F)	0.87	0.39	−1.17	0.85	0.10	33
18	*S. epidermidis* (E)	0.82	0.46	−0.88	0.83	0.10	36
19	*S. epidermidis* (F)	0.84	0.33	−1.45	0.76	0.08	32
20	*S. aureus* (E)	0.93	0.41	−1.06	1.01	−0.01	86
21	*S. aureus* (F)	0.96	0.36	−1.36	0.90	−0.06	49
22	*S. aureus* MRSA (E)	0.78	0.25	−1.18	1.17	−0.07	23
23	*S. aureus* MRSA (F)	0.70	0.23	−1.53	1.06	−0.02	19
24	*E. hirae* (E)	0.91	0.33	−1.10	1.07	−0.03	43
25	*E. hirae* (F)	0.72	0.22	−1.53	1.05	−0.01	19
26	*P. vulgaris* (E)	0.68	0.36	−1.15	0.68	0.11	39
27	*K. pneumoniae* (E)	0.68	0.38	−0.83	0.57	0.20	31
28	*K. pneumoniae* (F)	0.80	0.23	−1.11	0.75	0.09	23
29	*C. albicans* (E)	0.81	0.47	−1.25	1.00	0.00	57
30	*C. albicans* (F)	0.68	0.30	−1.37	0.86	0.02	24
31	*R. rubra* (E)	0.64	0.39	−0.84	0.76	0.15	41
32	*R. rubra* (F)	0.59	0.34	−1.31	0.75	0.06	24
33	*S. enteritidis* (E)	0.67	0.11	−0.79	2.22	2.15	21
34	*S. enteritidis* (F)	0.72	0.10	−0.92	2.31	2.48	21
35	*L.monocytogenes* (E)	0.72	0.10	−1.06	2.55	3.14	21
36	*L.monocytogenes* (F)	0.64	0.11	−1.12	2.32	2.76	21
37	*E. faecalis* (E)	0.95	0.30	−1.14	1.09	−0.01	21
38	*C. glabrata* (E)	0.92	0.39	−1.43	1.01	−0.06	19
39	*C. tropicalis* (E)	0.90	0.45	−1.52	0.90	−0.15	19
40	*S. cerevisiae* (E)	0.86	0.51	−1.25	0.92	−0.11	21
41	*S. cerevisiae JG* (E)	0.88	0.47	−1.24	0.96	−0.09	20
42	*S. cerevisiae JGCDR1* (E)	0.92	0.38	−1.31	0.94	−1.05	20
43	*G. candidum* (E)	0.88	0.47	−1.14	1.13	0.00	20
44	*R. mucilaginosa* (E)	0.93	0.41	−1.17	1.06	−0.03	21
45	ǂAcetylcholinesterase (A)	0.07	0.65	0.82	0.19	0.71	226
46	ǂ*L. Minor* (A)	0.34	0.57	0.52	0.95	0.02	10
47	ǂ*P. subcupitata* (A)	0.17	0.67	0.45	0.58	0.13	26
48	ǂ*P. vulgaris* (F)	0.31	0.31	−1.74	0.24	0.06	27
49	ǂ*P. aeruginosa* (E)	0.32	0.32	−1.77	0.41	−0.11	34
50	ǂ*P. aeruginosa* (F)	0.01	0.20	−1.90	−0.04	−0.38	15
51	ǂ*S. marcescens* (E)	0.19	0.41	−1.41	0.31	0.08	28
52	ǂ*S. marcescens* (F)	0.11	0.33	−1.72	0.24	−0.11	22
53	GR^III^ of *B. paxillifer* (A)	0.82	0.22	2.30	0.65	0.79	10
54	GR^III^ of *G. amphibium* (A)	0.98	0.16	2.81	1.43	−1.19	10
55	GR^III^ of *C. vulgaris* (A)	0.92	0.32	0.46	1.56	−0.23	10
56	GR^III^ of *O. submarina* (A)	0.95	0.22	0.10	1.38	−0.02	10
57	GR^III^ of *S. marinoi* (A)	0.88	0.32	2.73	1.15	−0.41	10
58	GR^III^ of *C. meneghiniana* (A)	0.91	0.22	2.41	0.97	0.08	10

And coefficient of determination (R^2^) and standard error (SE) by Eq. (4) and the determined sensitivity-related terms (z_x_, α_x_, and β_x_).

^ǂ^exceptional cases that comprehensive model does not cover^I^, A – Log 1/EC_50_, B – Log 1/LC_50_, C – Log 1/IC_50_, E-Log 1/MIC, F-Log 1/MBC^II^, Photosynthetic activity^III^, Growth rate.
