# Supplementary material for: Comprehensive approach for predicting toxicological effects of ionic liquids on several biological systems using unified descriptors
Source: Sci Rep. 2016 Sep 14;6:33403. doi: 10.1038/srep33403 (PMC5022054; doi:10.1038/srep33403)
Supplement: Supplementary Information 2 [file srep33403-s2.pdf]

Supplementary information 1

Comprehensive approach for predicting toxicological effects of ionic liquids on several biological systems using unified descriptors

Chul-Woong Cho[a], Stefan Stolte[b,c,*], Yeoung-Sang Yun[a,*]

[a]School of Chemical Engineering, Chonbuk National University, 567 Beakje-dearo, Deokjin-gu, Jeonju, Jeonbuk 561-756, Republic of Korea

[b]Centre for Environmental Research and Sustainable Technology (UFT), University of Bremen, Leobener Straße, 28359 Bremen, Germany

[c]Department of Environmental Analysis, Faculty of Chemistry, University of Gdańsk, ul.Wita Stwosza 63, 80-308 Gdańsk, Poland

Corresponding author – Tel: +82-63-270-2308 (Y.-S. Yun); fax: +82-63-270-2306(Y.-S. Yun); E-mail address: ysyun@jbnu.ac.kr (Y.-S. Yun), Tel: +49 421-218-63370 (S. Stolte); fax: 0049 421-218-98-63370(S. Stolte); E-mail: stefan.stolte@uni-bremen.de (S. Stolte)

Table S1. The abbreviations of (A) cation and (B) anion

(A) Abbreviations of Cations

Abbreviation of cation	Name
[Gu011112]$^+$	Tetramethylguanidinium
[HPiPy]$^+$	1-hexyl-4-piperidinopyridinium
[IM]$^+$	Imidazolium
[IM01]$^+$	1-methylimidazolium
[IM0-10]$^+$	1-decylimidazolium
[IM0-11]$^+$	1-undecylimidazolium
[IM0-12]$^+$	1-dodecylimidazolium
[IM01O-10]$^+$	1-(decyloxymethyl)imidazolium
[IM01O-11]$^+$	1-(undecyloxymethyl)imidazolium
[IM01O-12]$^+$	1-(dodecyloxymethyl)imidazolium
[IM01O-4]$^+$	1-(butoxymethyl)imidazolium
[IM01O-5]$^+$	1-(pentoxymethyl)imidazolium
[IM01O-6]$^+$	1-(hexyloxymethyl)imidazolium
[IM01O-7]$^+$	1-(heptoxymethyl)imidazolium
[IM01O-8]$^+$	1-(decyloxymethyl)imidazolium
[IM01O-9]$^+$	1-(nonyloxymethyl)imidazolium
[IM02Cl]$^+$	1-(2-ethylchloro)imidazolium
[IM04]$^+$	1-butylimidazolium
[IM06]$^+$	1-hexylimidazolium
[IM07]$^+$	1-heptylimidazolium
[IM08]$^+$	1-octylimidazolium
[IM09]$^+$	1-nonylimidazolium
[IM1-(1Ph-4Me)]$^+$	1-methyl-3-(phenylmethyl)imidazolium
[IM1-10]$^+$	1-decyl-3-methylimidazolium
[IM1-12]$^+$	1-dodecyl-3-methylimidazolium
[IM1-14]$^+$	1-methyl-3-tetradecylimidazolium
[IM1-16]$^+$	1-octadecyl-3-methylimidazolium
[IM1-18]$^+$	1-hexadecyl-3-methylimidazolium
[IM11CN]$^+$	1-(cyanomethyl)-3-methylimidazolium
[IM11O2]$^+$	1-(ethoxymethyl)-3-methylimidazolium
[IM1-1Ph]$^+$	1-methyl-3-(phenylmethyl)imidazolium
[IM12]$^+$	1-ethyl-3-methylimidazolium
[IM12=1]$^+$	1-methyl-3-(2-propenyl)imidazolium
[IM1-2C6F13]$^+$	1-methyl-3-(3,3,4,4,5,5,6,6,7,7,8,8,8-tridecafluorooctyl)imidazolium
[IM12Cl]$^+$	1-(2-chloroethyl)-3-methylimidazolium
[IM1-2CO-1]$^+$	1-methyl-3-(3-oxobutyl)imidazolium

$[IM12O1]^+$	3-(2-methylethyl)-3-methylimidazolium
$[IM12O2]^+$	1-(2-ethoxyethyl)-3-methylimidazolium
$[IM12OH]^+$	1-(2-hydroxyethyl)-3-methylimidazolium
$[IM1-2Ph]^+$	1-methyl-3-(2-phenylethyl)imidazolium
$[IM13]^+$	1-methyl-3-propylimidazolium
$[IM13Cl]^+$	1-(3-chloropropyl)-3-methylimidazolium
$[IM13COOH]^+$	3-(3-carboxypropyl)-1-methylimidazolium
$[IM13O1]^+$	1-(3-methoxypropyl)-3-methylimidazolium
$[IM13OH]^+$	1-(3-hydroxypropyl)-3-methylimidazolium
$[IM14]^+$	1-butyl-3-methylimidazolium
$[IM14-2Me]^+$	1-butyl-2,3-dimethylimidazolium
$[IM14OH]^+$	1-(4-hydroxybutyl)-3-methylimidazolium
$[IM15]^+$	1-methyl-3-pentylimidazolium
$[IM16]^+$	1-hexyl-3-methylimidazolium
$[IM16-2Me]^+$	1-hexyl-2,3-dimethylimidazolium
$[IM17]^+$	1-heptyl-3-methylimidazolium
$[IM17COOH]^+$	1-(7-carboxyheptyl)-3-methylimidazolium
$[IM18]^+$	1-methyl-3-octylimidazolium
$[IM18-2Me]^+$	1-octyl-2,3-dimethylimidazolium
$[IM18OH]^+$	1-(8-hydroxyoctyl)-3-methylimidazolium
$[IM19]^+$	1-methyl-3-nonylimidazolium
$[IM2-10]^+$	1-decyl-3-ethylimidazolium
$[IM23]^+$	1-ethyl-3-propylimidazolium
$[IM24]^+$	1-ethyl-3-butylimidazolium
$[IM26]^+$	1-ethyl-3-hexylimidazolium
$[Mel]^+$	Melamine
$[Mor11CN]^+$	4-(cyanomethyl)-4-methylmorpholinium
$[Mor11O2]^+$	4-(ethoxymethyl)-4-methylmorpholinium
$[Mor12]^+$	4-ethyl-4-methylmorpholinium
$[Mor12O1]^+$	4-(2-methoxyethyl)-4-methylmorpholinium
$[Mor12O2]^+$	4-(2-ethoxyethyl)-4-methylmorpholinium
$[Mor12OH]^+$	4-(2-hydroxyethyl)-4-methylmorpholinium
$[Mor13O1]^+$	4-(3-methoxypropyl)-4-methylmorpholinium
$[Mor13OH]^+$	4-(3-hydroxypropyl)-4-methylmorpholinium
$[Mor14]^+$	4-butyl-4-methylmorpholinium
$[MPS2Pip]^+$	1-methyl-1-[4,5- bis(methylsulfide)pentyl]piperidinium
$[MPS2Pyr]^+$	1-methyl-1-[4,5- bis(methylsulfide)pentyl]pyrrolidinium
$[N00(2O1)2O1]^+$	Bis(2-methoxyethyl)ammonium
$[N00(2O2)2]^+$	Bis(2-methoxyethyl)ammonium
$[N0112OH]^+$	2-(dimethylamino)ethanol

$[N0112OH]^+$	(2-hydroxyethyl)dimethylammonium
$[N11\text{-}10\text{-}1Ph]^+$	Benzyldecyldimethylammonium
$[N1111]^+$	Tetramethylammonium
$[N111\text{-}12]^+$	Dodecyltrimethylammonium
$[N111\text{-}14]^+$	Tetradecyltrimethylammonium
$[N111\text{-}16]^+$	Hexadecyltrimethylammonium
$[N11\text{-}12\text{-}1Ph]^+$	Benzyldodecyldimethylammonium
$[N1112O1]^+$	Methoxycholine
$[N1112OH]^+$	(2-hydroxyethyl)trimethylammonium
$[N1114]^+$	Butyltrimethylammonium
$[N11\text{-}14\text{-}1Ph]^+$	Benzyltetradecyldimethylammonium
$[N1121CN]^+$	(Cyanomethyl)ethyldimethylammonium
$[N1121COO2]^+$	(Ethoxycarbonylmethyl)ethyldimethylammonium
$[N1121O2]^+$	(Ethoxymethyl)ethyldimethylammonium
$[N1122O1]^+$	Ethyl(2-methoxyethyl)dimethylammonium
$[N1122O2]^+$	(2-ethoxyethyl)ethyldimethylammonium
$[N1122OH]^+$	Ethyl(2-hydroxyethyl)dimethylammonium
$[N1123]^+$	Ethyldimethylpropylammonium
$[N1123O1]^+$	Ethyl(3-methoxypropyl)dimethylammonium
$[N1123OH]^+$	Ethyl(3-hydroxypropyl)dimethylammonium
$[N1124]^+$	butylethyldimethylammonium
$[N112OH\text{-}1Ph]^+$	benzyl(2-hydroxyethyl)dimethylammonium
$[N112OH\text{-}2{=}1]^+$	(2-hydroxyethyl)dimethyl(2-propylene)ammonium
$[N112OH\text{-}4]^+$	(2-hydroxyethyl)dimethylbutylammonium
$[N112OH\text{-}8]^+$	(2-hydroxyethyl)dimethyloctylammonium
$[N1222]^+$	Triethylmethylammonium
$[N1888]^+$	Trioctylmethylammonium
$[N2221\text{-}Ph]^+$	(1-phenylmethyl)triethylammonium
$[N2222]^+$	Triethylammonium
$[N2222{=}1]^+$	Triethyl(2-propenyl)ammonium
$[N2224]^+$	Butyltriethylammonium
$[N2226]^+$	Hexyltriethylammonium
$[N2228]^+$	Octyltriethylammonium
$[N4444]^+$	Tetrabutylammonium
$[P1i4i4i4]^+$	Methyltris(2-methylpropyl)phosphanium
$[P2444]^+$	Tributylethylphosphonium
$[P2666]^+$	Ethyltrihexylphosphonium
$[P3666]^+$	Trihexylpropylphosphonium
$[P444\text{-}14]^+$	Tributyltetradecylphosphonium
$[P4444]^+$	Tetrabutylphosphonium

$[P4666]^+$	Butyltrihexylphosphonium
$[P666\text{-}10]^+$	Decyltrihexylphosphonium
$[P666\text{-}12]^+$	Dodecyltrihexylphosphonium
$[P666\text{-}14]^+$	Trihexyltetradecylphosphonium
$[P666\text{-}16]^+$	Hexadecyltrihexylphosphonium
$[P6666]^+$	Tetrahexylphosphonium
$[P6667]^+$	Heptyltrihexylphosphonium
$[P6668]^+$	Trihexyloctylphosphanium
$[Pip11CN]^+$	1-(cyanomethyl)-1-methylpiperidinium
$[Pip11O2]^+$	1-(ethoxymethyl)-1-methylpiperidinium
$[Pip12O1]^+$	1-(2-methoxyethyl)-1-methylpiperidinium
$[Pip12O2]^+$	1-(2-ethoxyethyl)-1-methylpiperidinium
$[Pip12OH]^+$	1-(2-hydroxyethyl)-1-methylpiperidinium
$[Pip13]^+$	1-methyl-1-propylpiperidinium
$[Pip13O1]^+$	1-(3-methoxypropyl)-1-methylpiperidinium
$[Pip13OH]^+$	1-(3-hydroxypropyl)-1-methylpiperidinium
$[Pip14]^+$	1-butyl-1-methylpiperidinium
$[Pip14CN]^+$	1-(4-cyanobutyl)-1-methylpiperidinium
$[Pip16]^+$	1-hexyl-1-methylpiperidinium
$[Pip18]^+$	1-octyl-1-methylpiperidinium
$[Pip24]^+$	1-butyl-1-ethylpiperidinium
$[Pip28]^+$	1-octyl-1-ethylpiperidinium
$[Py0]^+$	Pyridinium
$[Py1\text{-}4NMe2]^+$	4-(dimethylamino)-1-methylpyridinium
$[Py1\text{-}Bz]^+$	1-(phenylmethyl)pyridinium
$[Py1CN]^+$	1-(cyanomethyl)pyridinium
$[Py1O\text{-}10\text{-}3CONH2]^+$	3-(aminocarbonyl)-1-[(decyloxy)methyl]pyridinium
$[Py1O\text{-}11\text{-}3CONH2]^+$	3-(aminocarbonyl)-1-[(undecyloxy)methyl]pyridinium
$[Py1O\text{-}11\text{-}3OH]^+$	3-hydroxy-1-[(undecyloxy)methyl]pyridinium
$[Py1O\text{-}11\text{-}4CONH2]^+$	4-(aminocarbonyl)-1-[(undecyloxy)methyl]pyridinium
$[Py1O\text{-}12\text{-}3CONH2]^+$	3-(aminocarbonyl)-1-[(dodecyloxy)methyl]pyridinium
$[Py1O\text{-}12\text{-}4CONH2]^+$	4-(aminocarbonyl)-1-[(decyloxy)methyl]pyridinium
$[Py1O2]^+$	1-(ethoxymethyl)pyridinium
$[Py1O3\text{-}3OH]^+$	3-hydroxy-1-(propoxymethyl)pyridinium
$[Py1O4\text{-}3OH]^+$	1-(butoxymethyl)-3-hydroxypyridinium
$[Py1O6\text{-}3OH]^+$	1-[(hexyloxy)methyl]-3-hydroxypyridinium
$[Py1O7\text{-}3OH]^+$	1-[(heptyloxy)methyl]-3-hydroxypyridinium
$[Py2]^+$	1-ethylpyridinium
$[Py2{=}1]^+$	1-(2-propylene)pyridinium
$[Py2\text{-}4NMe2]^+$	4-(dimethylamino)-1-ethylpyridinium

$[Py2O1]^+$	1-(2-methoxyethyl)pyridinium
$[Py2O2]^+$	1-(2-ethoxyethyl)pyridinium
$[Py2OH]^+$	1-(2-hydroxyethyl)pyridinium
$[Py3]^+$	1-propylpyridinium
$[Py3\text{-}3Me]^+$	3-methyl-1-propylpyridinium
$[Py3O1]^+$	1-(3-methoxypropyl)pyridinium
$[Py3OH]^+$	1-(3-hydroxypropyl)pyridinium
$[Py3SO3H]^+$	1-(3-sulfopropyl)pyridinium
$[Py4]^+$	1-butylpyridinium
$[Py4\text{-}2Me]^+$	1-butyl-2-methylimidazolium
$[Py4\text{-}2Me\text{-}3Me\text{-}5Me]^+$	1-butyl-2,3,5-trimethylimidazolium
$[Py4\text{-}3Me]^+$	1-butyl-3-methylimidazolium
$[Py4\text{-}3Me\text{-}4Me]^+$	1-butyl-3,4-dimethylimidazolium
$[Py4\text{-}3Me\text{-}5Me]^+$	1-butyl-3,5-dimethylimidazolium
$[Py4\text{-}4Me]^+$	1-butyl-4-methylimidazolium
$[Py4\text{-}4NMe2]^+$	1-butyl-4-(dimethylamino)pyridinium
$[Py5]^+$	1-pentylpyridinium
$[Py6]^+$	1-hexylpyridinium
$[Py6\text{-}3Me]^+$	1-hexyl-3-methylpyridinium
$[Py6\text{-}3Me\text{-}4NMe2]^+$	1-hexyl-3-methyl-4-(dimethylamino)pyridinium
$[Py6\text{-}4Me]^+$	1-hexyl-4-methylpyridinium
$[Py6\text{-}4NMe2]^+$	4-(dimethylamino)-1-hexylpyridinium
$[Py8]^+$	1-octylpyridinium
$[Py8\text{-}2Me]^+$	2-methyl-1-octylpyridinium
$[Py8\text{-}3Me]^+$	3-methyl-1-octylpyridinium
$[Py8\text{-}4Me]^+$	4-methyl-1-octylpyridinium
$[Pyr11CN]^+$	1-(cyanomethyl)-1-methylpyrrolidinium
$[Pyr11COO2]^+$	1-(2-ethoxy-2-oxoethyl)-1-methylpyrrolidinium
$[Pyr11O2]^+$	1-(ethoxymethyl)-1-methylpyrrolidinium
$[Pyr12O1]^+$	1-(2-methoxyethyl)-1-methylpyrrolidinium
$[Pyr12O2]^+$	1-(2-ethoxyethyl)-1-methylpyrrolidinium
$[Pyr12OH]^+$	1-(2-hydroxyethyl)-1-methylpyrrolidinium
$[Pyr13]^+$	1-methyl-1-propylpiperidinium
$[Pyr13O1]^+$	1-(3-methoxypropyl)-1-methylpyrrolidinium
$[Pyr13OH]^+$	1-(3-hydroxypropyl)-1-methylpyrrolidinium
$[Pyr14]^+$	1-butyl-1-methylpyrrolidinium
$[Pyr16]^+$	1-hexyl-1-methylpyrrolidinium
$[Pyr18]^+$	1-methyl-1-octylpyrrolidinium
$[Pyr66]^+$	1,1-dihexylpyrrolidinium
$[Quin8]^+$	1-octylquinolinium

$[S122]^+$	Diethylmethylsulfonium
$[S222]^+$	Triethylsulfonium
$[TMG]^+$	Tetramethylguanidinium
$[TMSiMmIM]^+$	1-methyl-3-(trimethylsily)methyl-imidazolium
$[Xn1111]^+$	1-Purinium

(B) Abbreviations of Anions

Abbreviation of anion	Name
$[(2\text{-}OPhO)_2B]^-$	Bis[1,2-benzenediolato(2-)]borate
$[HO1(1)COO\_S]^-$	(2S)-2-hydroxypropanoate
$[(2\text{-}SO2PhCO)N]^-$	1,1-dioxo-1,2-dihydrobenzo[d]isothiazol-3-onate
$[HO1(1)COO]^-$	2-hydroxypropanoate
$[Cl_4SO_3]^-$	4-chloro-1-butanesulfonate
$[F_4SO_3]^-$	4-fluoro-1-butanesulfonate
$[4MePhSO_3]^-$	4-methylbenzenesulfonate
$[AC]^-$	6-methyl-2,2-dioxo-1,2,3-oxathiazin-4-onate
$[1COO]^-$	Acetate
$[(244Me3Pen)_2PO_2]^-$	Bis(2,4,4-trimethylpentyl)phosphinate
$[(C_2F_5)_2PO_2]^-$	Bis(pentafluoroethyl)phosphinate
$[(CF_3)_2N]^-$	Bis(trifluoromethyl)amide
$[(CF_3SO_2)_2N]^-$	Bis(trifluoromethylsulfonyl)amide
$[(OOCCOO)_2B]^-$	Bis[oxalato(2-)]borate
Br^-	Bromide
$[Cap]^-$	Caprylate
Cl^-	Chloride
$[9COO]^-$	Decanoate
$[Dep]^-$	Diethyl phosphate
$[2OSO_3]^-$	Ethylsulphate
$[HCOO]^-$	Formate
$[Gly]^-$	Glycinate
$[SbF_6]^-$	Hexafluoroantimonate
$[PF_6]^-$	Hexafluorophosphate
$[HSO_4]^-$	Hydrogen sulfate
$[HO1COO]^-$	Hydroxyacetate
I^-	Iodide
$[Ala]^-$	L-alaninate
$[Arg]^-$	L-argininate
$[Asp]^-$	L-asparaginate
$[Glu]^-$	L-glutaminate

$[His]^-$	L-histidinate
$[Iso]^-$	L-isoleucinate
$[Leu]^-$	L-leucinate
$[Lys]^-$	L-lysinate
$[Met]^-$	L-methioninate
$[Phe]^-$	L-phenylanine
$[Pro]^-$	L-prolinate
$[Ser]^-$	L-serinate
$[Thr]^-$	L-threoninate
$[Try]^-$	L-tryptophan
$[Val]^-$	L-valinate
$[1OSO_3]^-$	Methylsulphate
$[N(CN)_2]^-$	N-cyanocyanamide
$[NO_3]^-$	Nitrate
$[1O_2O_2OSO_3]^-$	*O*-2-(2-methoxyethoxy)ethyl sulfate
$[8OSO_3]^-$	Octylsulphate
$[pTs]^-$	*p*-Toluenesulfonate
$[N00SO_3]^-$	Sulfamate
$[B(CN)_4]^-$	Tetracyanidoboranuide
$[BF_4]^-$	Tetrafluoroborate
$[SCN]^-$	Thiocynate
$[(C_3F_7)_3PF_3]^-$	Trifluoridotris(heptafluoropropyl)phosphate
$[(C_2F_5)_3PF_3]^-$	Trifluoridotris(pentafluoroethyl)phosphate
$[CF_3COO]^-$	Trifluoroacetate
$[CF_3SO_3]^-$	Trifluoromethansulfonate
$[(CF_3SO_2)_3C]^-$	Tris(trifluoromethylsulfonyl)methanide

Explanations on sub-parameters

The sub-parameters are polar surface area (PSA), COSMO-volume (V_c), energy of van der Waals (E_{vdw}), molar refraction (MR), sigma moments ($\sigma_{1\text{-}6}$), hydrogen-bond donor ($HBD_{1\text{-}4}$) and hydrogen-bond acceptor abilities ($HBA_{1\text{-}4}$). The sigma moments describe polarization of charge density on its molecule surface; σ_1: negative of total charge, σ_2: overall electrostatic polarity of a solute, σ_3: asymmetry of the sigma profile, $\sigma_{4\text{-}6}$: no simple physical interpretation; the σ_5 has an approximate linear relationship with σ_3, while σ_4 and σ_6 have asymmetry of the σ_3. The hydrogen bond moments are quantitative measurements of the acceptor and donor of the molecule, and they can be defined in the same way as sigma moments (Eckert 1999-2014).

Calculation models for LFER descriptors using sub-parameters

The obtained sub-parameters based on obprop, DFT and COSMO were used to calculate LFER descriptors. Here, molecular weight/100 (MW), COSMO-volume/100 (V_c), molecular weight and sigma moments (1-6) were rescaled by dividing 100. Additionally, we used the number of ring (N_{Ring}), number of OH group (N_{OH}) and number of hydrogen atom attached to nitrogen (N_{HN}). The calculation models (as shown below) were previously presented by our group (Cho et al. 2015).

**V [(cm³mol⁻¹)/100]**	$= 0.639\ (0.002)\ \mathbf{V_{cosmo}}\ [nm^3] - 0.0046\ (0.002)$	**Eq.(S1)**
**E [cm³mol⁻¹/10]**	$= 0.341\ \mathbf{N_{Ring}} + 0.007\ \mathbf{PSA} + 0.057\ \mathbf{MR} - 1.762\ \mathbf{V_{cosmo}/100} - 0.113\ \mathbf{E_{vdw}} + 0.275\ \mathbf{MW} + 0.135\boldsymbol{\sigma_1} + 0.015\ \boldsymbol{\sigma_4} - 0.037$	**Eq.(S2)**

R^2=0.949, SE=0.136, N=992, F=2274.8

**A [dimensionless]**	$= 171\ \mathbf{HBD_1^2} - 0.047\ \mathbf{HBD_3^2} + 0.032\ \mathbf{HBD_4^2} + 73.511\ \mathbf{HBD_1} + 0.654\ \mathbf{HBD_2} + 0.208\mathbf{HBD_3} + 0.203\mathbf{N_{OH}} + 0.080\ \mathbf{N_{HN}} - 0.019\ \mathbf{E_{vdw}} - 0.132$	**Eq.(S3)**

R^2=0.936, SE=0.148, N=976, F=1626.2

**B[dimensionless]**	$= 0.391\boldsymbol{\sigma_2} + 1.00\ \boldsymbol{\sigma_3} + 0.421\ \boldsymbol{\sigma_4} - 0.117\ \boldsymbol{\sigma_5} - 0.055\ \boldsymbol{\sigma_6} + 0.112\ \boldsymbol{\sigma_1^2} - 0.149\ \boldsymbol{\sigma_2^2} - 0.070\ \mathbf{HBA_2/V_{cosmo}} + 0.074\ \mathbf{HBA_3/V_{cosmo}} + 0.032$	**Eq.(S4)**

R^2=0.973, SE=0.160, N=985, F=3851

$$J^{+}[\text{dimensionless}] = -0.124(0.234) - 0.106(0.016)\,\mathbf{E_{vdw}} + 0.421(0.181)\,\boldsymbol{\sigma_3} + 0.292(0.125)\,\boldsymbol{\sigma_4} + 64.928(15.48)\,\mathbf{HBD_1} + 0.661(0.163)\,\mathbf{HBD_2} - 0.049(0.017)\,\mathbf{HBA_2/V_C} - 0.092(0.033)\,\boldsymbol{\sigma_6}^{2}/\mathbf{100} \quad \text{Eq.(S5)}$$

R^2=0.816, SE=0.351, N=111, F=65.4

$$J^{-}[\text{dimensionless}] = 1.331(0.468) + 4.712(0.803)\,\boldsymbol{\sigma_2} - 2.770(0.262)\,\boldsymbol{\sigma_3} - 0.832(0.168)\,\boldsymbol{\sigma_2}^{2} + 0.300(0.040)\,\boldsymbol{\sigma_3}^{2} - 0.012(0.004)\,\boldsymbol{\sigma_4}^{2} - 0.155(0.021)\,\mathbf{HBA_2} + 0.238(0.023)\,\mathbf{HBA_3} - 0.292(0.141)\,\mathbf{N_{OH}} + 0.183(0.065)\,\mathbf{N_{Ring}} \quad \text{Eq.(S6)}$$

R^2=0.711, SE=0.291, N=168, F=43.2

$$S\,[\text{dimensionless}] = -1.441\,\boldsymbol{\sigma_1} + 0.206\,\boldsymbol{\sigma_2}^{2} - 0.009\,\boldsymbol{\sigma_4}^{2} - 0.122\,\mathbf{HBA_4} + 0.511\,\textit{Calc.E} + 1.524\,\mathbf{Calc.\,B} + 0.856\,\mathbf{Calc.\,J^{+}} + 3.308\,(\boldsymbol{\sigma_1}\mathbf{*Calc.J^{-})/Calc.B} - 0.099 \quad \text{Eq.(S7)}$$

R^2=0.940, SE=0.378, N=981, F=1900.7

Table S2. (Example case) Z-setting method for determining system parameters (e_c, s_c, a_c, b_c, v_c, j^-, e_a, s_a, a_a, b_a, v_a, j^+, and c) and z_1~z_4 value of toxicity testing system in multiple linear regression analysis

	Toxicity values	LFER Descriptors												System 1	System 2	System 3	System 4
		E_c	S_c	A_c	B_c	V_c	J^+	E_a	S_a	A_a	B_a	V_a	J^-	Z_1	Z_2	Z_3	Z_4
System 1	Insert experimentally determined toxicity values e.g., log 1/EC_{50} (mM)	Insert calculated LFER descriptor values of ILs												1	0	0	0
														1	0	0	0
														1	0	0	0
														1	0	0	0
System 2														0	1	0	0
														0	1	0	0
														0	1	0	0
														0	1	0	0
System 3														0	0	1	0
														0	0	1	0
														0	0	1	0
														0	0	1	0
System 4														0	0	0	1
														0	0	0	1
														0	0	0	1
														0	0	0	1

Figure S1. Correlations between calculated by Eq. (4) and observed toxicity values of ILs

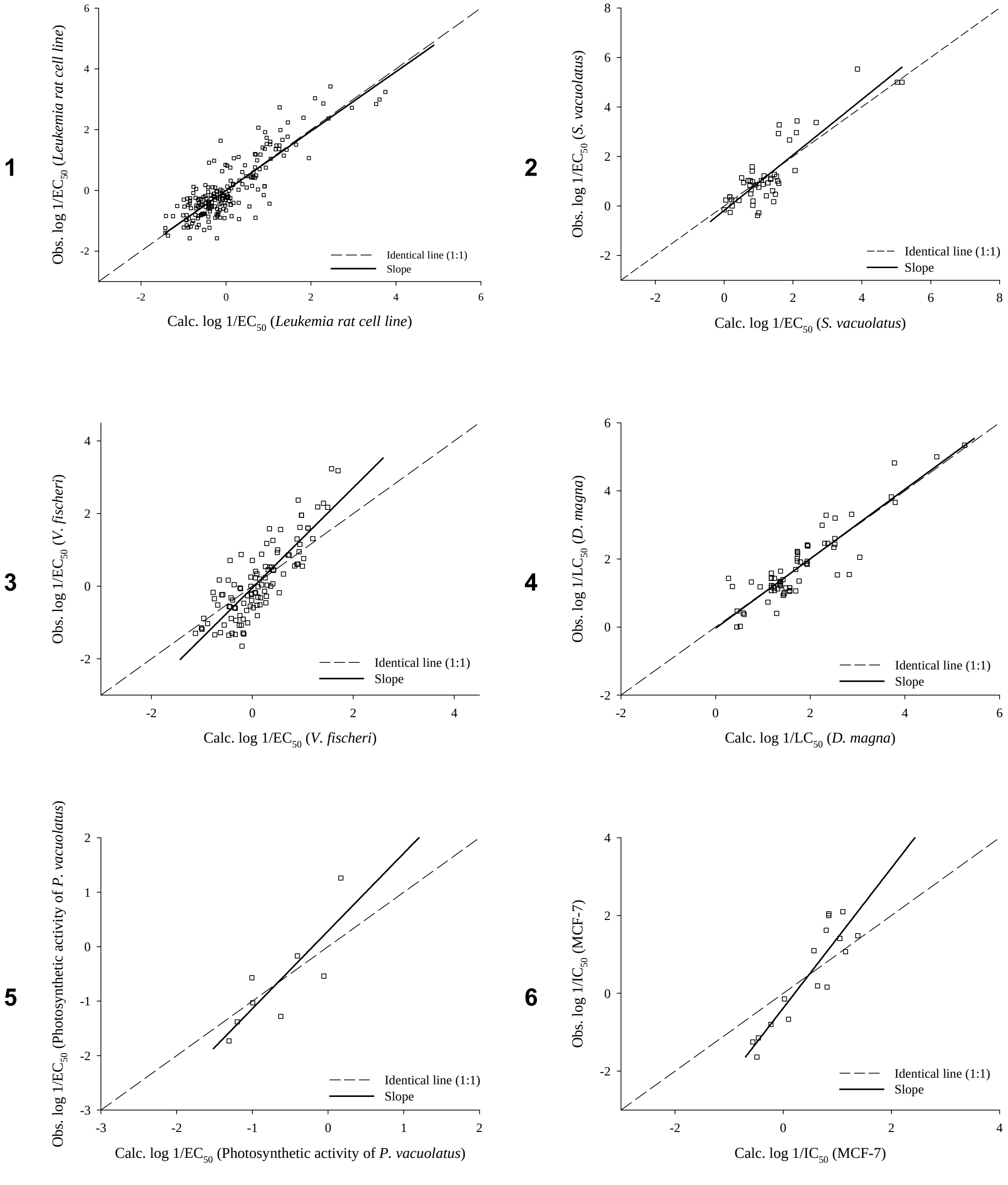

7

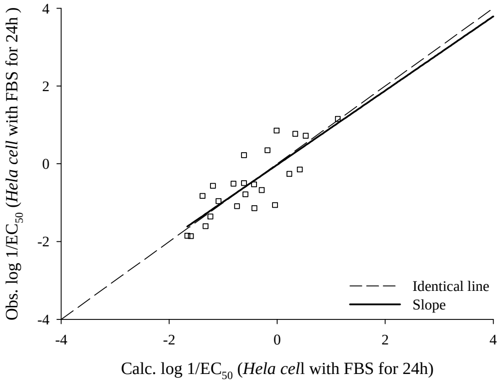

Obs. log 1/EC50 (Hela cell with FBS for 24h)
Calc. log 1/EC50 (Hela cell with FBS for 24h)
Identical line
Slope

8

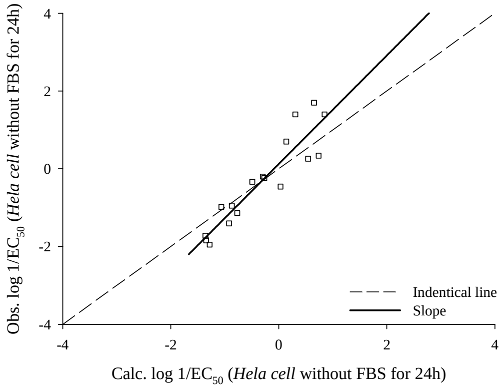

Obs. log 1/EC50 (Hela cell without FBS for 24h)
Calc. log 1/EC50 (Hela cell without FBS for 24h)
Indentical line
Slope

9

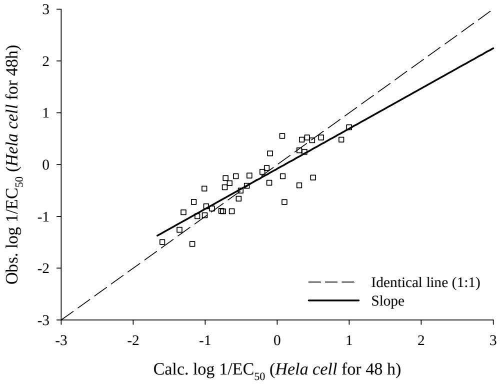

Obs. log 1/EC50 (Hela cell for 48h)
Calc. log 1/EC50 (Hela cell for 48 h)
Identical line (1:1)
Slope

10

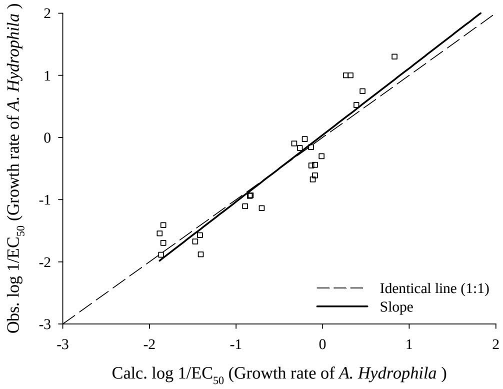

Obs. log 1/EC50 (Growth rate of A. Hydrophila)
Calc. log 1/EC50 (Growth rate of A. Hydrophila)
Identical line (1:1)
Slope

11

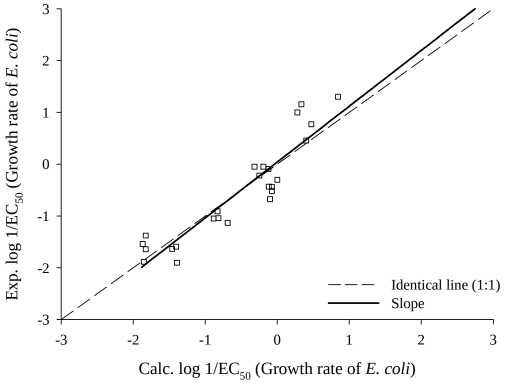

Exp. log 1/EC50 (Growth rate of E. coli)
Calc. log 1/EC50 (Growth rate of E. coli)
Identical line (1:1)
Slope

12

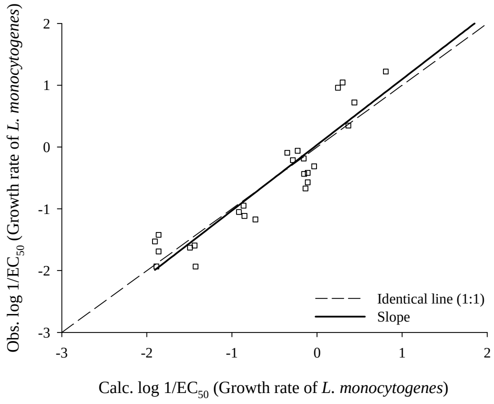

Obs. log 1/EC50 (Growth rate of L. monocytogenes)
Calc. log 1/EC50 (Growth rate of L. monocytogenes)
Identical line (1:1)
Slope

13

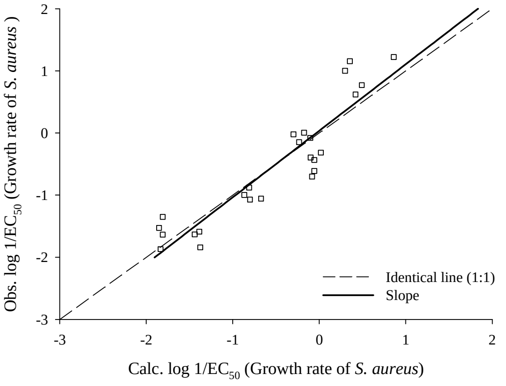

14

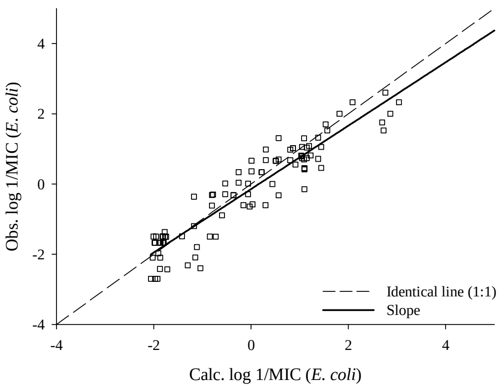

15

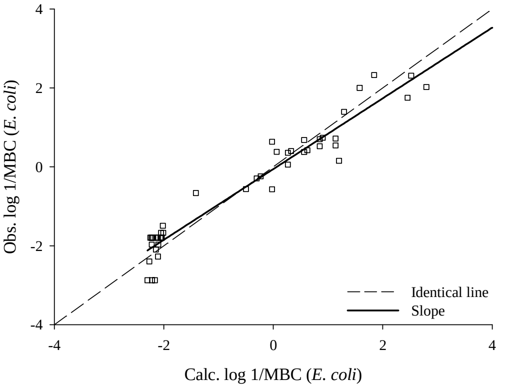

16

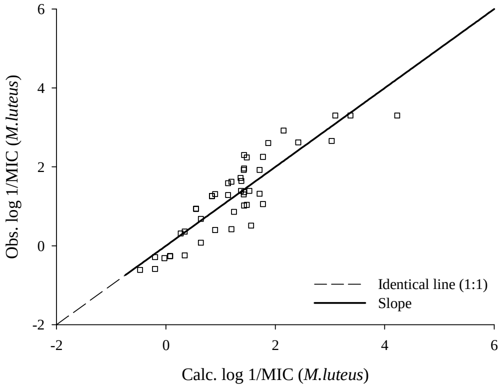

17

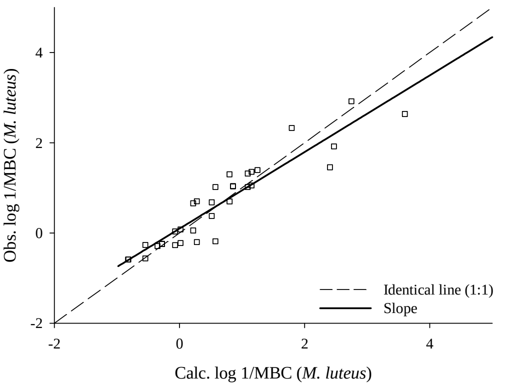

18

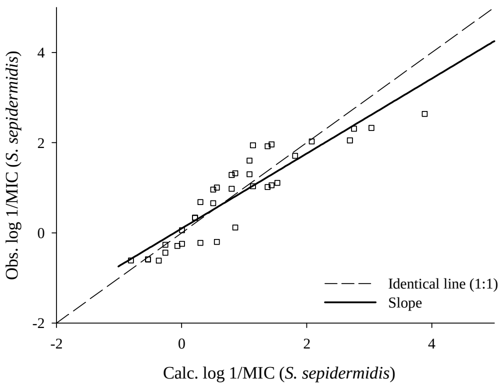

19

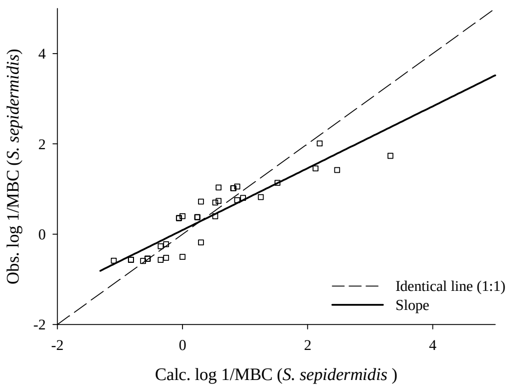

20

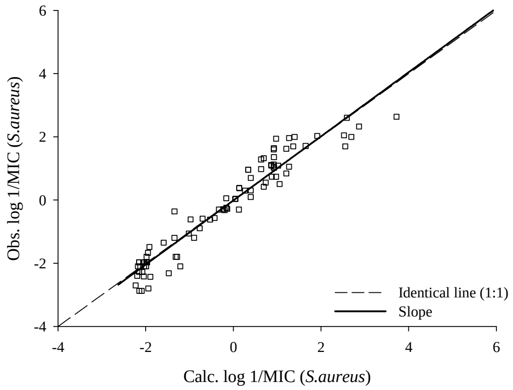

21

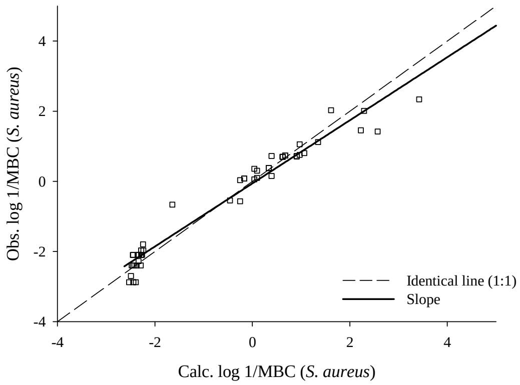

22

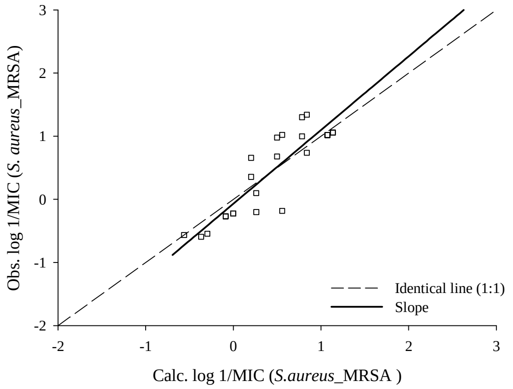

23

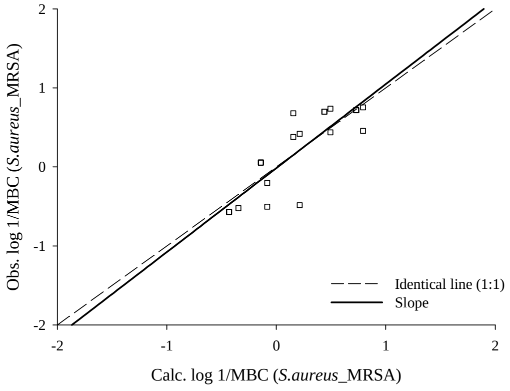

24

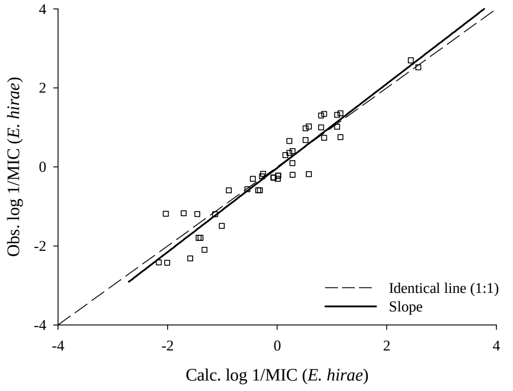

25

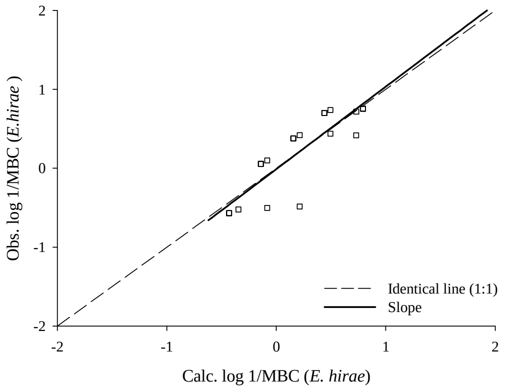

26

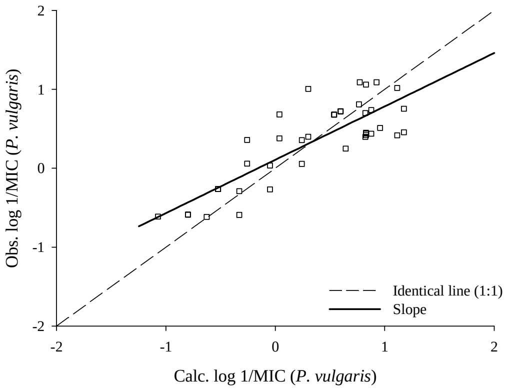

27

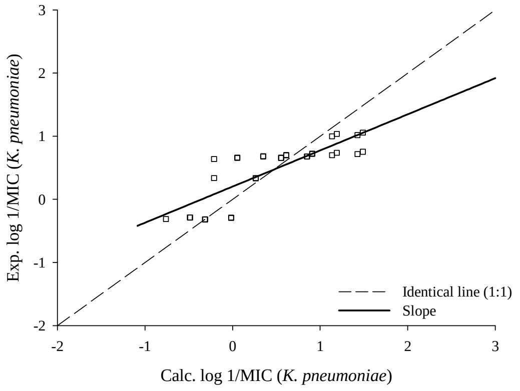

28

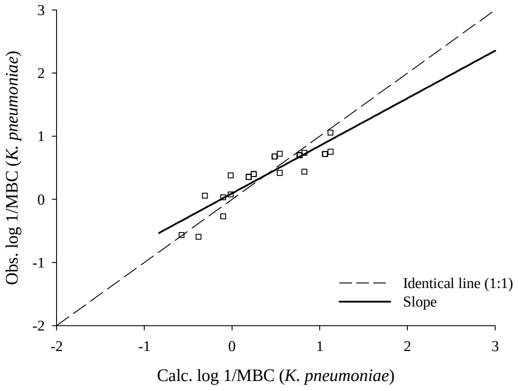

29

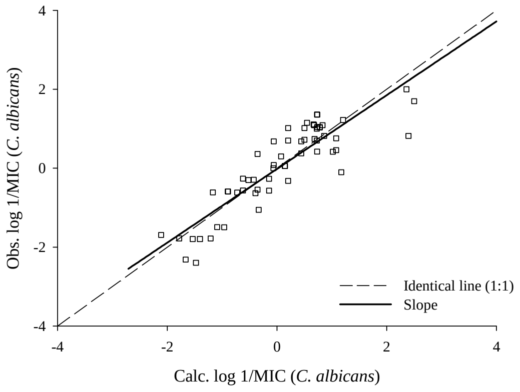

30

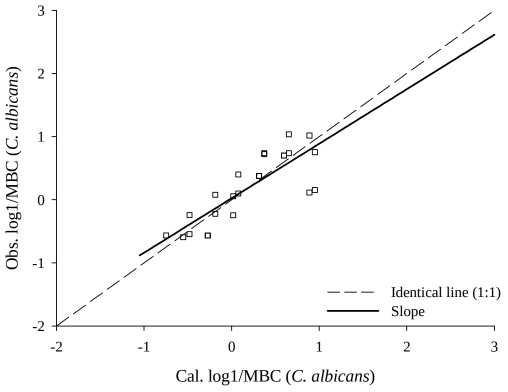

31

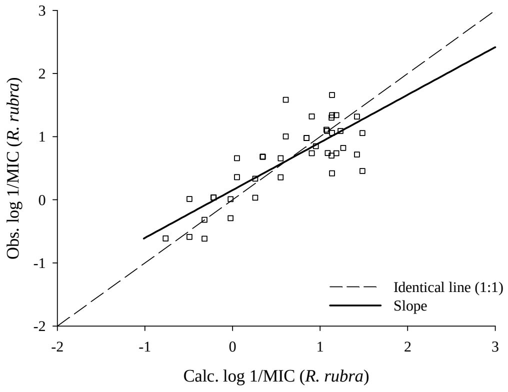

32

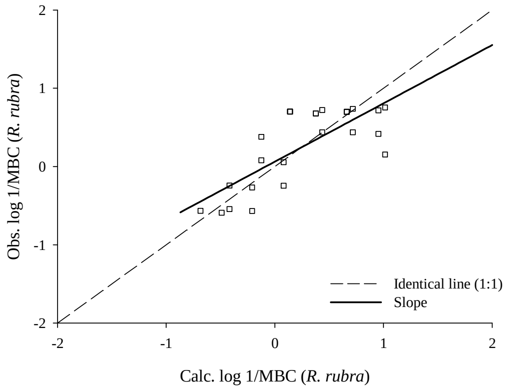

33

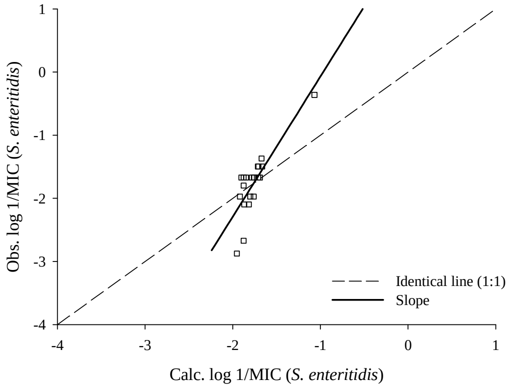

34

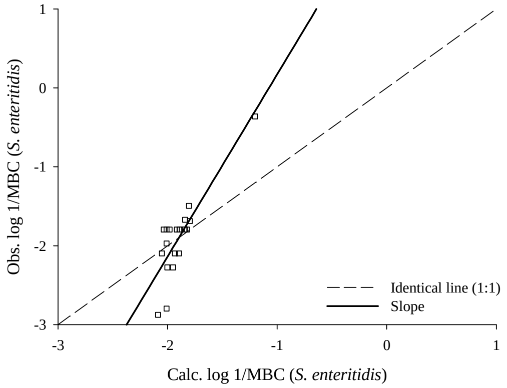

35

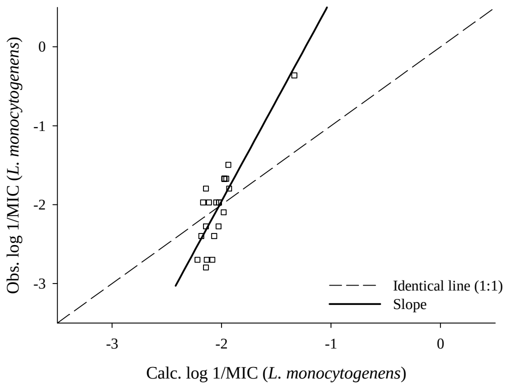

36

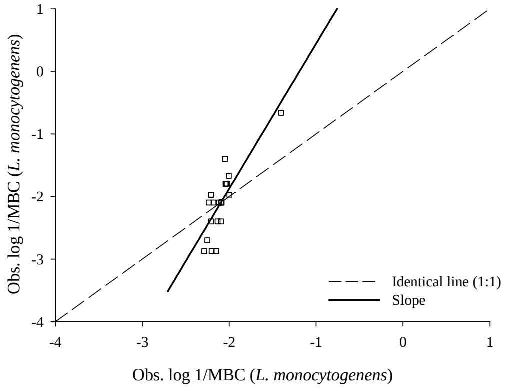

37

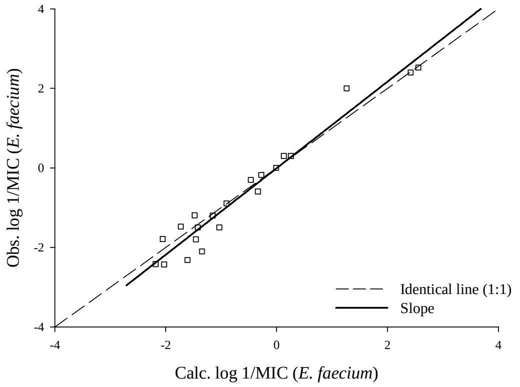

38

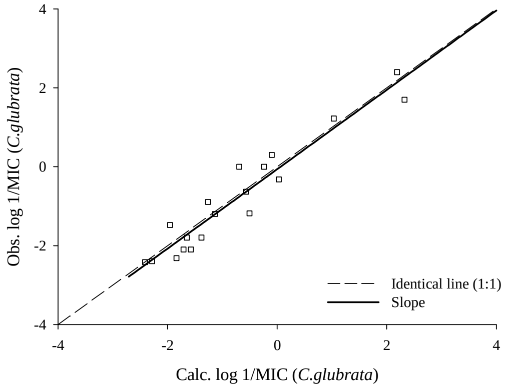

39

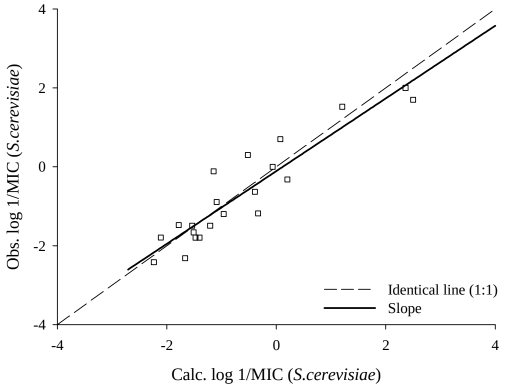

40

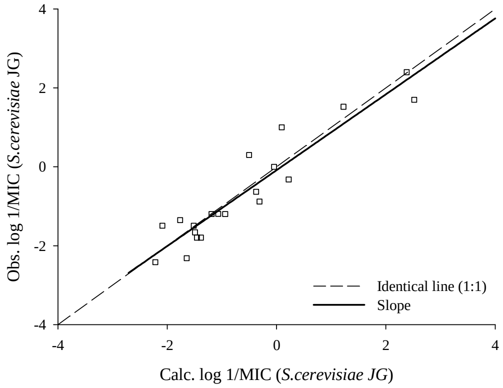

41

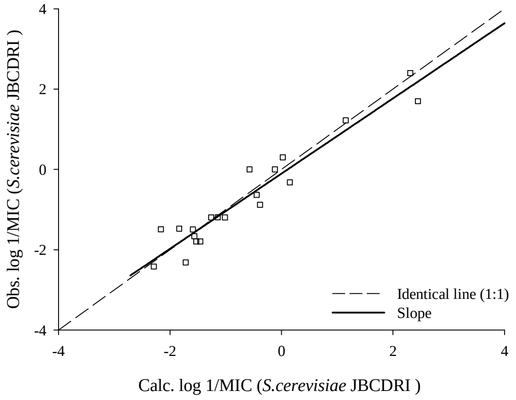

42

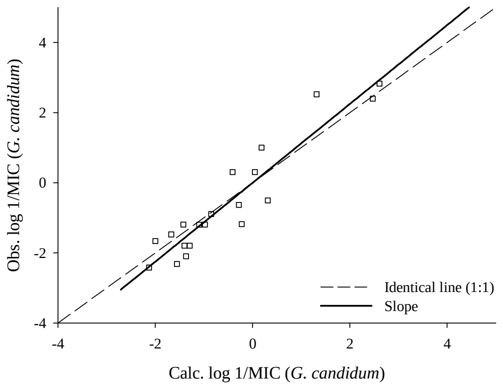

4
3

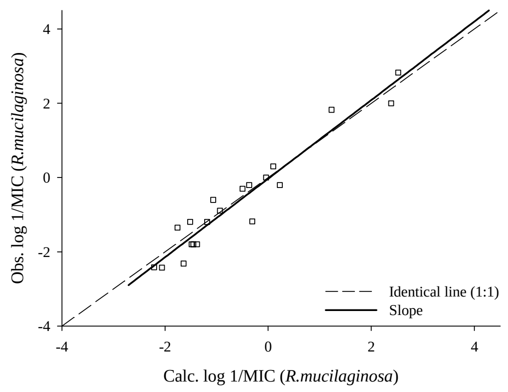

4
4

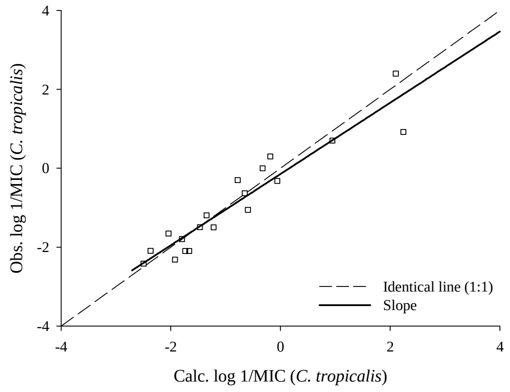

5
3

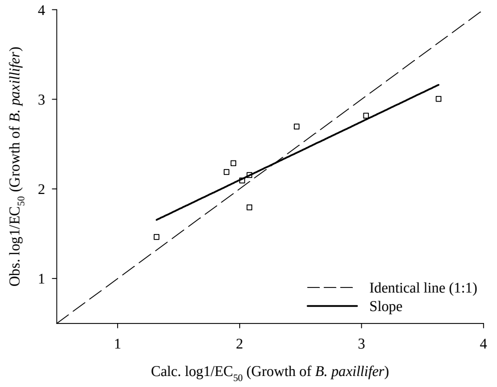

5
4

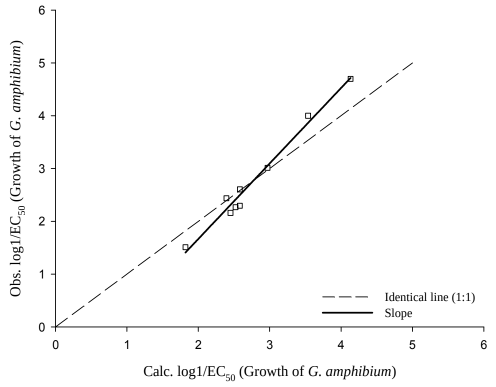

5
5

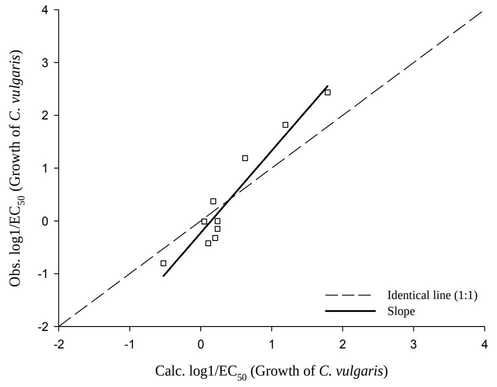

5
6

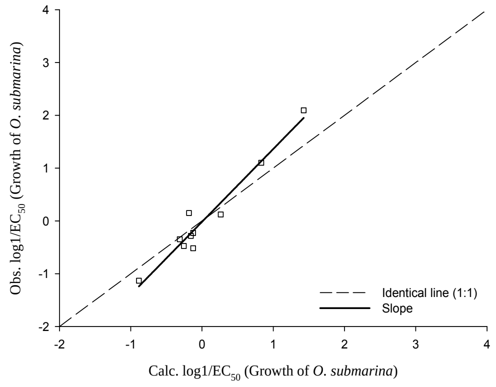

5
7

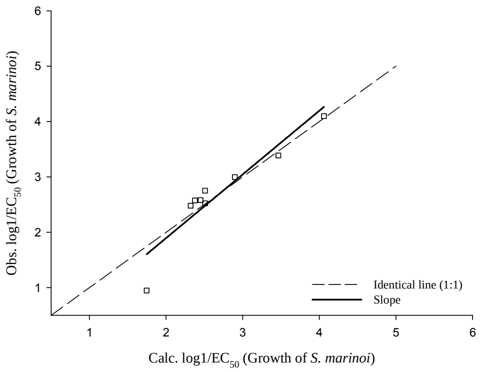

Obs. log1/EC50 (Growth of S. marinoi)
Calc. log1/EC50 (Growth of S. marinoi)
Identical line (1:1)
Slope

5
8

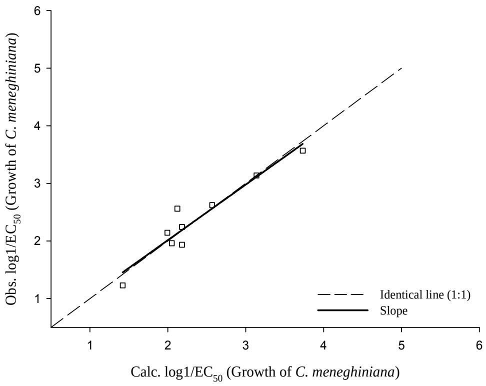

Obs. log1/EC50 (Growth of C. meneghiniana)
Calc. log1/EC50 (Growth of C. meneghiniana)
Identical line (1:1)
Slope

Figure S2. Correlations between calculated [by Eq. (4)] and observed ILs' log $1/EC_{50}$ (*Acetylcholinesterase*-45 and *L. minor*-46), log 1/MBC (*P. vulgaris*-48, *P. aureginosa*-50, and *S. marcesens*-52), and 1/MIC (*P. aureginosa*-49, and *S. marcesens*-51)

45

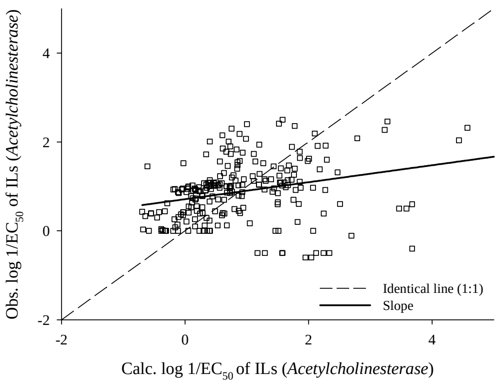

46

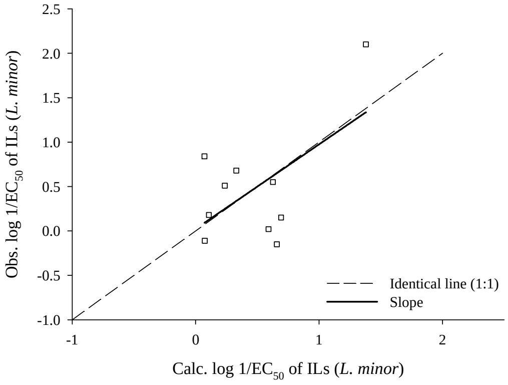

48

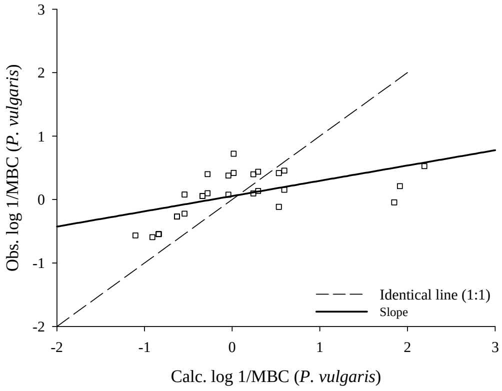

49

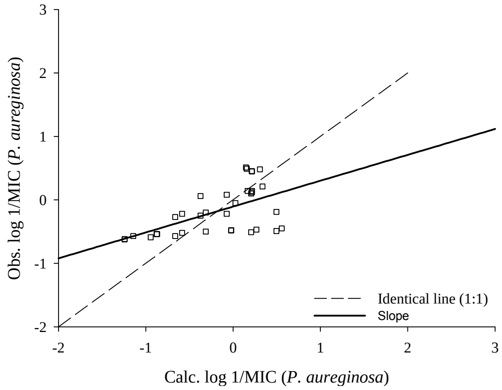

50

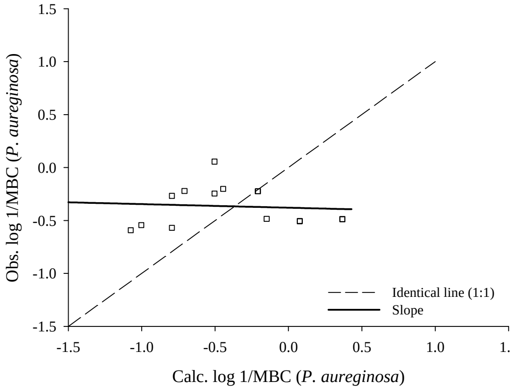

51

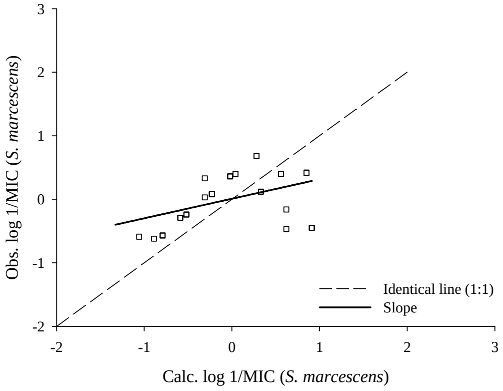

5

2

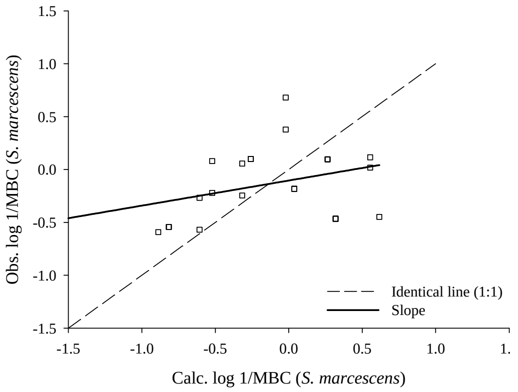
Obs. log 1/MBC (*S. marcescens*)
1.5
1.0
0.5
0.0
-0.5
-1.0
-1.5
Identical line (1:1)
Slope
-1.5
-1.0
-0.5
0.0
0.5
1.0
1.
Calc. log 1/MBC (*S. marcescens*)

Figure S3. A correlation between calculated [by Eq. (4)] and observed log 1/EC_{50} of ILs to *growth rate of P. subcapitata* (47)

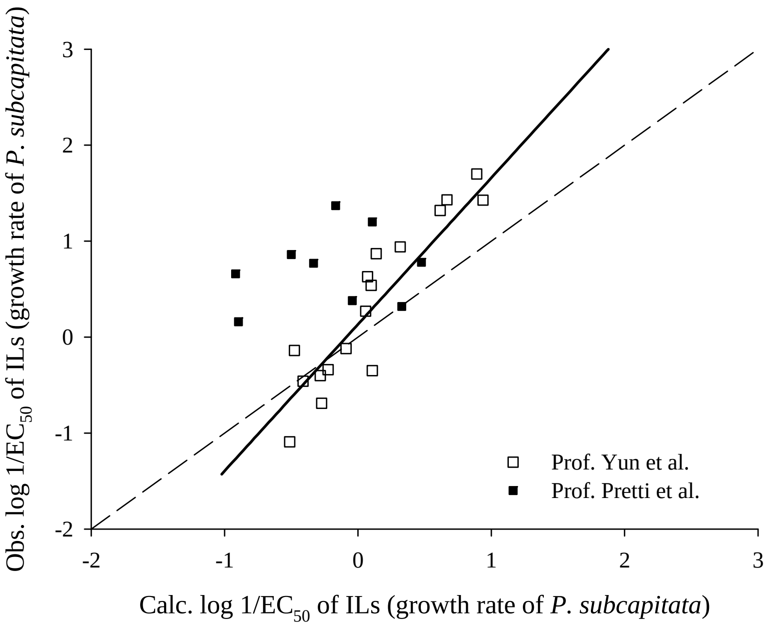

Table S3. The magnitude of anionic toxic effect calculated by an equation ($- 0.201\ E_a + 0.418\ V_a + 0.131\ J^-$)

Anion	Calculated value	Anion	Calculated value
I^-	0.12	$[His]^-$	0.57
$[Br]^-$	0.24	$[B(CN)_4]^-$	0.59
$[NO_3]^-$	0.25	$[Gln]^-$	0.59
$[SCN]^-$	0.29	$[Val]^-$	0.6
Cl^-	0.3	$[Arg]^-$	0.6
$[HCOO]^-$	0.3	$[PF_6]^-$	0.63
$[HSO_4]^-$	0.35	$[Met]^-$	0.64
$[1SO_3]^-$	0.36	$[1O2O2OSO_3]^-$	0.64
$[N(CN)_2]^-$	0.36	$[Leu]^-$	0.65
$[HO1COO]^-$	0.36	$[Phe]^-$	0.65
$[1COO]^-$	0.4	$[Iso]^-$	0.66
$[2OSO_3]^-$	0.42	$[Trp]^-$	0.68
$[N00SO_3]^-$	0.42	$[(2\text{-}OPhO)_2B]^-$	0.69
$[HO1(1)COO]^-$	0.42	$[Lys]^-$	0.69
$[AC]^-$	0.42	$[F_4SO_3]^-$	0.69
$[BF_4]^-$	0.43	$[Cl_4SO_3]^-$	0.71
$[Gly]^-$	0.44	$[Cap]^-$	0.74
$[Ser]^-$	0.45	$[(CF3)_2N]^-$	0.74
$[1OSO_3]^-$	0.46	$[8OSO_3]^-$	0.76
$[Pro]^-$	0.48	$[(C2F5)_2PO_2]^-$	0.8
$[Asp]^-$	0.49	$[Dep]^-$	0.8
$[CF_3SO_3]^-$	0.49	$[SbF_6]^-$	0.82
$[Thr]^-$	0.51	$[(CF_3SO_2)_2]^-$	0.82
$[(2\text{-}SO2PhCO)N]^-$	0.52	$[9COO]^-$	0.85
$[CF3COO]^-$	0.52	$[(CF_3SO_2)_3C]^-$	1.16
$[(OOCCOO)_2B]^-$	0.52	$[(244Me3Pen)_2PO_2]^-$	1.33
$[Glu]^-$	0.54	$[(C2F5)_3PF_3]^-$	1.4
$[Asn]^-$	0.55	$[(C3F7)_3PF_3]^-$	1.83
$[4MePhSO_3]^-$	0.57		

Table S4. The magnitude of cationic toxic effect calculated by an equation ($2.254\,E_c - 2.545\,S_c + 0.646\,A_c - 1.471\,B_c + 1.650\,V_c + 2.917\,J^{+\,-}$)

Name	Calc. toxicity value	Name	Calc. toxicity value
Gu011112	-1.08	IM01O-11	2.32
HPiPy	2.63	IM01O-12	2.62
IM	-0.27	IM01O-4	0.37
IM01	-0.49	IM01O-5	0.64
IM04	0.23	IM01O-6	0.92
IM1-10	1.73	IM01O-7	1.19
IM1-12	2.55	IM01O-8	1.48
IM1-14	2.86	IM01O-9	1.74
IM1-16	4.16	IM02Cl	0.73
IM1-18	4.02	IM06	0.82
IM11CN	-0.26	IM07	1.11
IM11O2	-0.17	IM08	1.39
IM1-1Ph	1.03	IM09	1.68
IM12	-0.58	IM1-(1Ph-4Me)	1.25
IM1-2C6F13	2.04	IM1-10	1.73
IM12O1	-0.83	IM1-12	2.30
IM12O2	-0.17	IM1-14	2.86
IM12OH	-0.61	IM1-16	4.16
IM13	-0.29	IM1-18	4.02
IM13O1	-0.96	IM11O2	-0.17
IM13OH	-0.77	IM1-1Ph	1.03
IM14	-0.01	IM12	-0.58
IM14-2Me	0.15	IM12=1	-0.25
IM15	0.28	IM12Cl	0.36
IM16	0.57	IM1-2CO-1	-0.39
IM16-2Me	0.57	IM12O1	-0.83
IM17	0.87	IM12O2	-0.17
IM18	1.14	IM12OH	-0.61
IM19	1.45	IM1-2Ph	1.12
IM24	0.18	IM13	-0.29
IM26	0.75	IM13Cl	0.07
Melamine	0.97	IM13COOH	-0.73
Mor11CN	0.44	IM13O1	-0.96
Mor11O2	-0.75	IM13OH	-0.77
Mor12	-0.44	IM14	-0.01

Mor12O1	-0.71	IM14OH	-0.87
Mor12O2	-0.49	IM16-2Me	0.57
Mor12OH	-0.41	IM17	0.87
Mor13O1	-0.64	IM17COOH	-0.12
Mor13OH	-0.59	IM18	1.14
Mor14	0.03	IM18OH	-0.01
N0,1,1,2OH	-0.12	IM19	1.45
N1,1,2,4	-0.37	IM2-10	2.44
N11-10-1Ph	2.82	IM23	-0.08
N1111	-0.97	IM24	0.18
N11-12-1Ph	3.37	IM26	0.75
N1112O1	-0.99	Mor14	0.03
N1114	-0.32	N(2O2)2	-0.84
N11-14-1Ph	3.94	N1110-Bz	2.82
N1121CN	-0.53	N1112OH	-0.86
N1121COO2	-0.78	N1123	-0.54
N1121O2	-0.30	N1123O1	-1.09
N1122O1	-1.11	N1123OH	-1.13
N1122O2	-0.74	N1124	-0.37
N1123O1	-1.09	N112OH-1	-0.86
N1123OH	-1.13	N112OH-1Ph	0.52
N1124	-0.37	N112OH-2	-0.52
N1124	-0.37	N112OH-2=1	-0.52
N1222	-0.74	N112OH-4	-0.43
N1888	4.21	N112OH-8	0.62
N2226	0.49	N2221-Ph	0.82
N4444	1.41	N2222	-0.63
P1i4i4i4	1.03	N2222=1	-0.33
P2444	1.10	N2224	-0.03
P666-14	4.78	N2228	1.06
Pip11CN	0.19	N4444	1.41
Pip11O2	0.19	Nh112OH	-0.12
Pip12O1	-0.32	Nhhh2OH	-1.00
Pip12O2	-0.05	P2666	2.90
Pip12OH	-0.26	P3666	3.18
Pip13O1	-1.02	P444-14	4.63
Pip13OH	-0.49	P4444	1.90
Pip14	0.06	P4666	3.45
Py0	-0.15	P666-10	5.16
Py1-4NMe2	-0.17	P666-12	5.80

Py1CN	-0.13	P666-14	6.35
Py1O2	-0.25	P666-16	6.93
Py2	-0.28	P6666	4.06
Py2-4NMe2	0.05	P6667	4.33
Py2O1	-0.60	P6668	5.19
Py2O2	-0.96	Pip14	0.06
Py2OH	-0.34	Py1CN	-0.13
Py3	-0.07	Py1O-10-3-CONH2	2.39
Py3O1	-0.51	Py1O-11-3-CONH2	2.39
Py3OH	-0.45	Py1O11-3OH	3.05
Py3SO3H	0.91	Py1O-11-4-CONH2	2.34
Py4	0.22	Py1O-12-3-CONH2	2.39
Py4-3Me	0.20	Py1O-12-4-CONH2	2.39
Py4-3Me-4Me	0.19	Py1O2	-0.25
Py4-3Me-5Me	0.29	Py1O3-3OH	3.05
Py4-4Me	0.15	Py1O4-3OH	1.04
Py4-4NMe2	0.59	Py1O6-3OH	1.42
Py5	0.50	Py1O7-3OH	1.71
Py6	0.79	Py1-Ph	1.03
Py6-3Me	0.79	Py2	-0.28
Py6-4Me	0.72	Py2=1	0.03
Py6-4NMe2	1.17	Py2-4NMe2	0.05
Py8	1.36	Py2O1	-0.60
Py8-3Me	1.36	Py2O2	-0.96
Py8-4Me	1.32	Py2OH	-0.34
Pyr11COO2	-0.94	Py3	-0.07
Pyr11O2	-0.01	Py3-3Me	-0.12
Pyr12O1	-0.70	Py3O1	-0.51
Pyr12O2	-0.06	Py3OH	-0.45
Pyr12OH	-0.30	Py3SO3H	0.91
Pyr13O1	-0.59	Py4	0.22
Pyr13OH	-0.54	Py4-2Me	0.34
Pyr14	-0.04	Py4-3Me-4Me	0.19
Pyr16	0.53	Py4-3Me-5Me	0.29
Pyr18	1.09	Py4-4Me	0.15
Pyr66	1.73	Py4-4NMe2	0.59
Quin8	2.57	Py5	0.50

S122	-0.51	Py6	0.79
S222	-0.45	Py6-3Me	0.79
TMG	-0.71	Py6-4Me	0.72
TMSiMmIM	0.32	Py6-4NMe2	1.17
Xn1111	-0.48	Py8	1.36
IM01	-0.49	Py8-2Me	1.62
IM0-10	1.98	Py8-3Me	1.36
IM0-11	2.27	Py8-4Me	1.32
IM0-12	2.56	Pyr11CN	0.11
IM01O-10	2.04	Pyr11O2	-0.01
IM01O-11	2.32	Pyr12O1	-0.70
IM01O-12	2.62	Pyr12O2	-0.06
IM01O-4	0.37	Pyr12OH	-0.30
IM01O-5	0.64	Pyr13	0.03
IM01O-6	0.92	Pyr13O1	-0.59
IM01O-7	1.19	Pyr13OH	-0.54
IM01O-8	1.48	Pyr14	-0.04
IM01O-9	1.74	Pyr16	0.53
IM02Cl	0.73	Pyr18	1.09
IM06	0.82	Pyr66	1.73

References

1 C.-W. Cho, S. Stolte, Y.-S. Yun, I. Krossing, J. Thöming, *RSC Adv.*, 2015, **5**, 80634-80642.

2 F. Eckert, (1999-2014) COSMOtherm reference manual, version C3.0, Release 15.01, Leverkusen, Germany
